# Exploring galectin interactions with human milk oligosaccharides and blood group antigens identifies BGA6 as a functional galectin-4 ligand

**DOI:** 10.1016/j.jbc.2024.107573

**Published:** 2024-07-14

**Authors:** Alejandro J. Cagnoni, Mora Massaro, Anabela M. Cutine, Ana Gimeno, Juan M. Pérez-Sáez, Montana N. Manselle Cocco, Sebastián M. Maller, Santiago Di Lella, Jesús Jiménez-Barbero, Ana Ardá, Gabriel A. Rabinovich, Karina V. Mariño

**Affiliations:** 1 Laboratorio de Glicómica Funcional y Molecular, Programa de Glicoinmunología, Instituto de Biología y Medicina Experimental (IBYME), Consejo Nacional de Investigaciones Científicas y Técnicas (CONICET), Buenos Aires, Argentina; 2 Laboratorio de Glicomedicina, Programa de Glicoinmunología, Instituto de Biología y Medicina Experimental (IBYME), Consejo Nacional de Investigaciones Científicas y Técnicas (CONICET), Buenos Aires, Argentina; 3 CIC bioGUNE, Derio, Bizkaia, Spain; 4 Instituto de Química Biológica, Ciencias Exactas y Naturales (IQUIBICEN-CONICET), Ciudad de Buenos Aires, Argentina; 5 Departamento de Química Biológica, Facultad de Ciencias Exactas y Naturales, Universidad de Buenos Aires, Ciudad de Buenos Aires, Argentina; 6 Ikerbasque, Basque Foundation for Science, Bilbao, Bizkaia, Spain; 7 Department of Organic & Inorganic Chemistry, Faculty of Science and Technology University of the Basque Country, EHU-UPV, Leioa, Spain; 8 Centro de Investigación Biomédica En Red de Enfermedades Respiratorias, Madrid, Spain; 9 Universidad Argentina de la Empresa (UADE), Instituto de Tecnología (INTEC), Ciudad de Buenos Aires, Argentina

**Keywords:** galectins, blood group antigens, human milk oligosaccharides, inflammation, interleukin-6

## Abstract

Galectins (Gals), a family of multifunctional glycan-binding proteins, have been traditionally defined as β-galactoside binding lectins. However, certain members of this family have shown selective affinity toward specific glycan structures including human milk oligosaccharides (HMOs) and blood group antigens. In this work, we explored the affinity of human galectins (particularly Gal-1, -3, -4, -7, and -12) toward a panel of oligosaccharides including HMOs and blood group antigens using a complementary approach based on both experimental and computational techniques. While prototype Gal-1 and Gal-7 exhibited differential affinity for type I *versus* type II Lac/LacNAc residues and recognized fucosylated neutral glycans, chimera-type Gal-3 showed high binding affinity toward poly-LacNAc structures including LNnH and LNnO. Notably, the tandem-repeat human Gal-12 showed preferential recognition of 3-fucosylated glycans, a unique feature among members of the galectin family. Finally, Gal-4 presented a distinctive glycan-binding activity characterized by preferential recognition of specific blood group antigens, also validated by saturation transfer difference nuclear magnetic resonance experiments. Particularly, we identified oligosaccharide blood group A antigen tetraose 6 (BGA6) as a biologically relevant Gal-4 ligand, which specifically inhibited interleukin-6 secretion induced by this lectin on human peripheral blood mononuclear cells. These findings highlight unique determinants underlying specific recognition of HMOs and blood group antigens by human galectins, emphasizing the biological relevance of Gal-4-BGA6 interactions, with critical implications in the development and regulation of inflammatory responses.

Previous studies highlighted the critical roles of human lectins and their glycosylated ligands as relevant biomarkers and therapeutic targets in a broad range of pathological conditions including chronic inflammation, infectious diseases, autoimmunity, fibrosis, and cancer (1, 2, 3, 4). While Siglecs and most C-type lectin receptors are transmembrane proteins present on the cell surface, galectins act both extracellularly and intracellularly, modulating different cellular functions through protein–glycan or protein–protein interactions (5, 6, 7, 8).

Structurally, galectins (Gals) are defined by an evolutionary-conserved common structural fold, a β-sandwich consisting of two antiparallel β-sheets forming a concave and a convex side, and a conserved carbohydrate recognition domain (CRD) (9, 10). They have been traditionally classified as “proto-type” galectins, presenting a monomeric CRD in dynamic equilibrium with its homodimer (Gal-1, -2, -5, -7, -10, −11-, −13, −14, and −15), “tandem-repeat” type galectins, with two CRDs in tandem connected by a linker peptide (Gal-4, -8, -9, and -12), and the chimera-type Gal-3 containing a CRD connected to a non-lectin N-terminal domain responsible for oligomerization and ligand cross-linking (5, 11). Through interactions with selected glycosylated receptors on the cell surface (8), galectins can mediate relevant processes such as immunomodulation, cell adhesion, inflammation, pathogen recognition, and tumor immunity (3, 5, 7, 12, 13). In contrast to their traditional definition as proteins with affinity for *N*-acetyllactosamine (LacNAc), individual members of the galectin family have shown preferential recognition for a unique set of glycoconjugates (14, 15). Notably, biochemical assessment of bi-CRD galectins such as Gal-4, and particularly Gal-12, has been limited, probably due to difficulties in their recombinant expression (16, 17).

Human milk oligosaccharides (HMOs) are major components of milk, with approximately 200 unconjugated and structurally diverse oligosaccharides reported to date (18, 19). HMOs present a lactose core at their reducing end, which can be further extended with type I (β-D-Gal (1–3)-GlcNAc) or type II (β-D-Gal (1–4)-GlcNAc) LacNAc repeating units; these disaccharides can also be modified by α(1–2), α(1–3), or α(1–4) fucose moieties, and α(2–3) or α(2–6)-sialylation (19, 20, 21). HMOs are only minimally digested in the gastrointestinal tract, and further beyond their role as prebiotics, they contribute to generate a healthy intestinal microbiota (22, 23, 24, 25, 26), modulate neonatal immunity in the infant gut (27, 28, 29) and act as soluble decoys to prevent pathogen invasion (19, 25, 30, 31, 32). Interestingly, these oligosaccharide structures have been shown to play protective roles in a broad range of infant and adult pathologies including immunoglobulin E-associated eczema (33), necrotizing enterocolitis (34), cow milk protein allergy, rotavirus-associated infections (35, 36, 37, 38), asthma (39), as well as memory and learning disabilities (40). However, despite their significant roles in health promotion and disease prevention (19), the mechanisms by which HMOs control immune responses and intestinal homeostasis are still uncertain. Previous studies using shotgun glycan microarrays showed that human Gal-1, -3, -4, -7, -8, and -9 present selective HMO binding profiles, confirmed by isothermal titration calorimetry (ITC) and hapten inhibition experiments (41). In turn, analyses of the HMO-binding activity of human Gal-1, the C-terminal fragment of human Gal-3, human Gal-7, and the N-terminal fragment of human Gal-9 (Gal-9N) were also performed using electrospray ionization mass spectrometry (ESI-MS) (42). Understanding the molecular determinants underlying galectin-ligand recognition may help understand the immunoregulatory roles of HMOs in pathophysiological contexts.

In addition to HMOs, ABO-H blood antigens have also been evaluated as galectin ligands. These polymorphic carbohydrate structures are terminally exposed portions of larger glycans linked to proteins or lipids on the surface of erythrocytes, endothelial, and epithelial cells (43). Interestingly, during evolution, diverse microorganisms acquired the capacity of displaying blood group antigens on their surface, leading to molecular mimicry between pathogen and host cells (44). Specifically, Gal-3, -4, and -8 recognize blood group A and B antigens with high affinity on glycan microarrays and were able to kill microbes that express blood group-like determinants (45, 46, 47). The antimicrobial activity of Gal-7 has recently been demonstrated, and cooperative binding interactions were shown to be key players in this process, as Gal-7 exhibits relatively little binding activity to blood group A and blood group B-containing glycans (48, 49).

Given our interest in intestinal glycans, the relevance of HMOs in intestinal barrier function (50), and considering the ability of Gal-3, -4, and -7 to bind blood group A and B antigens, specifically recognizing blood group-like determinants expressing microbes (45, 46, 47, 49), we explored the molecular bases of these galectin-glycan interactions. Moreover, given the immunomodulatory role of Gal-1 in gut immune cell homeostasis (51), our previous work on murine Gal-12 (14) and the scarce data published on the human Gal-12 ortholog, we also evaluated HMOs and blood group antigens as potential ligands for these lectins. Thus, using competitive solid-phase assays (SPAs) and ITC, as well as *in silico* computational simulations, we successfully expanded previous data on Gal-1, -3, and -7, and provided new insights on tandem-repeat type human Gal-4 and -12. In this regard, we characterized human Gal-12, validating the preferential affinity for 3-fucosylated glycans previously described for the murine ortholog (14). Interestingly, our results show that blood group A antigen tetraose 6 (BGA6) is a high affinity, selective, and functional ligand for Gal-4 as determined by saturation transfer difference nuclear magnetic resonance (STD-NMR) experiments as well as a dose-dependent inhibitor of interleukin (IL)-6 production induced by Gal-4 on human peripheral blood mononuclear cells (PBMCs). It is tempting to speculate that Gal-4-BGA6 interactions may contribute to modulation of IL-6-dependent inflammatory responses in a wide range of immune-mediated pathologies, including autoimmune disorders, chronic infection, fibrosis and cancer.

## Results

### Competitive SPAs reveal distinct binding preferences of human galectins for HMOs and blood group antigens

**Figure 1:**
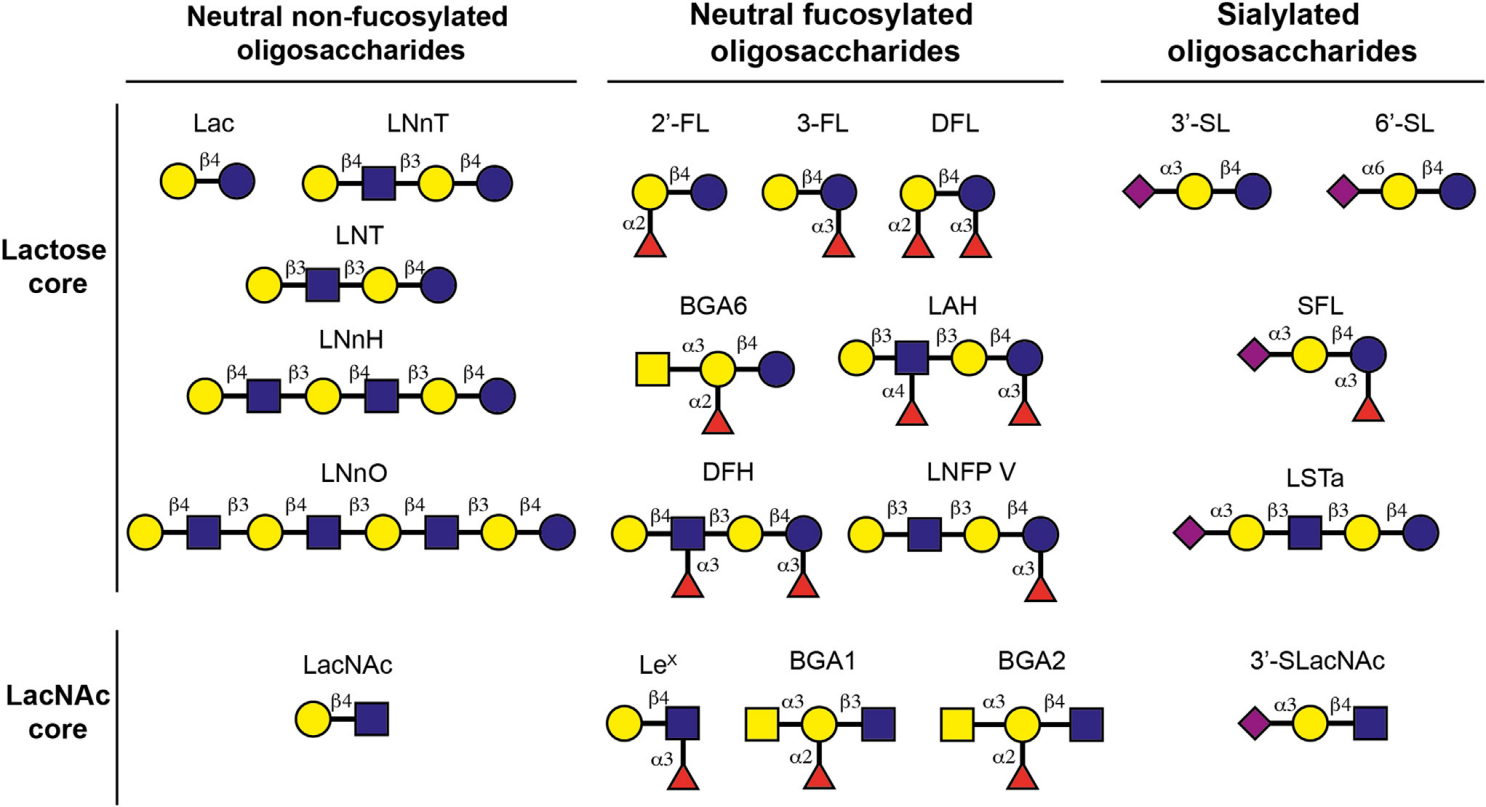
**Structure of selected oligosaccharides screened as potential galectin ligands.** Glycans are depicted following the current version of the Symbol Nomenclature for Glycans (SNFG) (101).

Human Gal-1, -3, -4, -7, and -12 were recombinantly expressed, purified, and their activity and glycan-binding preferences were screened by competitive SPAs using a set of neutral nonfucosylated, neutral fucosylated, and sialylated oligosaccharides. These included HMOs (based on a lactose core), and a group of glycan structural motifs based on the LacNAc core, including blood group antigens (BGAs) and Lewis X trisaccharide (Fig. 1).

**Figure 2:**
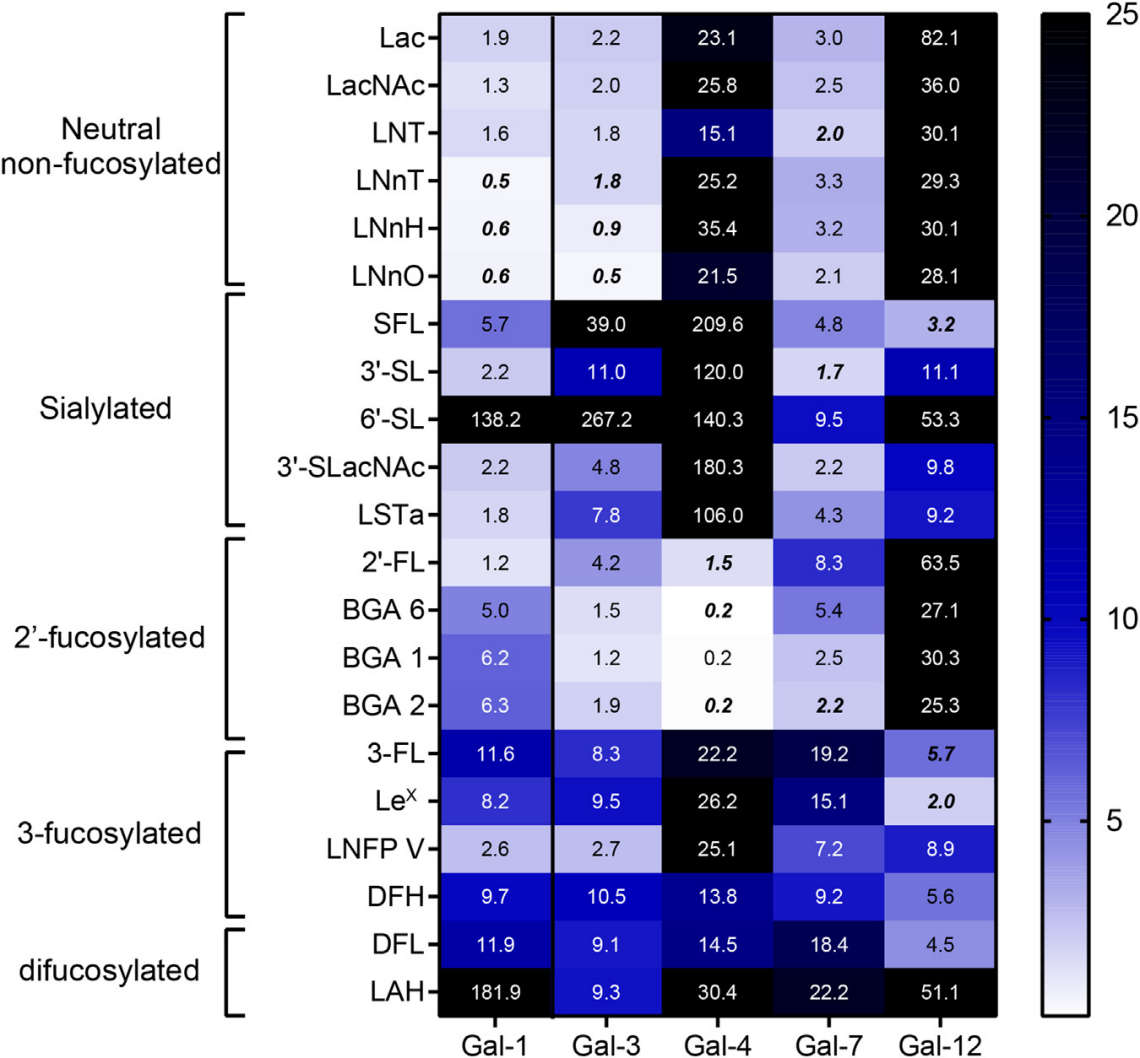
**Heatmap summarizing preferential binding of human galectin (Gal)-1, -3, -4, -7, and -12 by competitive solid phase assays (SPA) using immobilized asialofetuin.** Competitive interactions of galectins with a panel of 21 oligosaccharides, colored according to their IC_50_ values. Best binders are colored in *white* and low binders in *blue*. Glycans with IC_50_ values higher than 25 mM (poor ligands) are shown in *black*. The oligosaccharides selected for ITC analysis are marked in *bold* and *italics*. ITC, isothermal titration calorimetry.

Each individual member of the galectin family exhibited a specific glycan-binding preference (Figs. 2, S1, Table S1).

While human Gal-1 presented preferential binding toward oligosaccharides bearing (terminal) type II LacNAc (Gal*β* (1–4)GlcNAc) residues, such as lacto-*N*-neotetraose (LNnT), compared to type I LacNAc residues (Gal*β* (1–3)GlcNAc) like lacto-*N*-tetraose (LNT) tetrasaccharide, 6′-sialylation abrogated Gal-1 recognition, while 3′-sialylated structures presented moderate affinity. These results are consistent with previous findings by frontal affinity chromatography and glycan arrays (41, 48) (Figs. 2, S1, Table S1). Interestingly, among neutral nonfucosylated glycans, human Gal-1 showed lower affinity for LacNAc when compared to LNnT, while no significant improvement in binding affinity was observed for hexasaccharides and octasaccharides lacto-*N*-neohexaose (LNnH) and lacto-*N*-neooctaose (LNnO). Indeed, it has been recently described that Gal-1 only recognizes the terminal nonreducing LacNAc entity in a tri-LacNAc hexasaccharide (52). Considering fucosylated structures, human Gal-1 bound to 2′-FL with similar affinity to lactose, indicating that this fucose modification did not impact binding. However, the blood group glycans BGA2 and BGA6 (Fig. 1) displayed higher IC_50_ values than the parental Lac/LacNAc structures, showing that addition of terminal α(1–3)Gal/GalNAc hampers human Gal-1 affinity, consistent with previous NMR-based reports (53). When analyzing 3-fucosylated glycans (Fig. 1) affinity was considerably decreased (3-fucosyllactose (3-FL), Lewis X trisaccharide (Le^X^), difucosyllactose (DFL), and 2′,3-difucosylhexaose (DFH)) except for lacto-*N*-fucopentaose V (LNFP V) which showed moderate affinity, an effect probably associated to the terminal type I LacNAc moiety available for Gal-1 recognition. Finally, α(1–4) fucosylation abrogated Gal-1 binding, as demonstrated by Lewis A antigen hexaose (LAH) as a poor Gal-1 ligand.

Human Gal-3 showed preferential recognition of neutral HMOs, HMOs-related glycans and BGAs such as Lac, LacNAc, LNT, LNnT, LNnH, LNnO, BGA1, BGA2, and BGA6 (Figs. 2, S1, Table S1). In contrast, 3′- and 6′-sialylated structures, as well as 3-fucosylated structures, were poor competitors. Previous reports (54) have shown preferential binding of Gal-3 toward type II Lac/LacNAc residues compared to type I Lac/LacNAc units. In our competitive SPAs, the IC_50_ values for LNT and LNnT were similar (*ca*. 1.8 mM). In addition, Gal-3 showed increased binding with higher LacNAc units (IC_50_ values for LNnO<LNnH<LNnT<LacNAc, Fig. 2), consistent with its reported recognition of internal LacNAc units (48, 52).

In turn, tandem-repeat type human Gal-4 showed a marked preference for 2′-fucosylated blood group glycans, including 2′-FL, BGA6, BGA1, and BGA2 (Figs. 2, S1, Table S1). Glycan-binding preferences of full-length human Gal-4 aligned with those reported for its isolated CRDs (55, 56), with preferential binding toward BGA6. Our results are partially consistent with previous reports of human Gal-4-HMOs binding preferences (41); these differences could be attributed to glycan immobilization on shotgun microarray analysis as previously reported (57, 58). Finally, 3- or 4-fucosylated structures were not efficient competitors for Gal-4 binding, while 3′- and 6′-sialylation almost abrogated Gal-4 binding. Finally, neither 3-fucosylated structures (3-FL, Le^X^, and LNFP V) nor difucosylated LAH (which is both 3- and 4-fucosylated) were efficient competitors of Gal-4 binding, while 3′- and 6′-sialylation virtually abrogated Gal-4 binding.

Conversely, human Gal-7 recognized a broader spectrum of ligands among tested oligosaccharides, with preferential binding toward neutral nonfucosylated HMOs, but also blood group glycans (Figs. 2, S1, Table S1). Notably, Gal-7 binding was overall weaker as compared to Gal-1, -3, and -4. In contrast to human Gal-1, human Gal-7 exhibited certain increased binding for type I LacNAc residues (IC_50_ = 2.0 mM for LNT) compared to type II (IC_50_ = 3.3 mM for LNnT) similar to previous findings by glycan microarray and biolayer interferometry measurements (41, 54), while the increase in the number of LacNAc units from 2 (LNnT) to 3 (LNnH) did not affect affinity. This fact is also consistent with a certain preference (although not exclusive) of Gal-7 for the terminal nonreducing moiety of LacNAc repeats (52). The affinity of Gal-7 for these LacNAc-type structures was similar to that shown by Gal-1, which also belongs to the prototype family, although weaker (*ca.* 5-fold for LNnT, LNnH, and LNnO). Finally, 6′-sialylated glycans showed low affinity, while 3′-sialyllactose (3′-SL) was one of the best competitive inhibitors according to SPA assays. Moderate binding was also observed for 3′-SLacNAc and blood group antigens BGA1 and BGA2.

Finally, the glycan-binding affinities of human Gal-12 were evaluated. To obtain full-length human Gal-12, we adapted our previously optimized procedure for murine Gal-12 (14). In SPAs, human Gal-12 binding was weaker as compared to other members of the galectin family analyzed here, and showed low binding toward neutral oligosaccharides, including Lac and LacNAc. Similar to mouse Gal-12 (14), human Gal-12 preferentially recognized 3-fucosylated structures, including 3-FL and Le^X^ (Figs. 2, S1, Table S1). Further elongation of the 3-FL core with 3-fucosylated LacNAc did not alter human Gal-12 affinity, as demonstrated by 2′,3-difucosylhexaose binding (Figs. 2, S1, Table S1). However, 3-FL extension with α(1–4) fucosyl-LacNAc (as in LAH, Fig. 1) impaired human Gal-12 binding. Notably, DFL showed a comparable affinity to 3-FL as human Gal-12 ligand, indicating that α(1–2) fucosylation at the galactose residue of 3-FL does not significantly alter Gal-12 recognition. Finally, α(2–3) sialylation of Lac or LacNAc improved binding affinities when compared to parental disaccharides; however, this modification decreased human Gal-12 affinity for 3-fucosylated structures.

**Figure 3:**
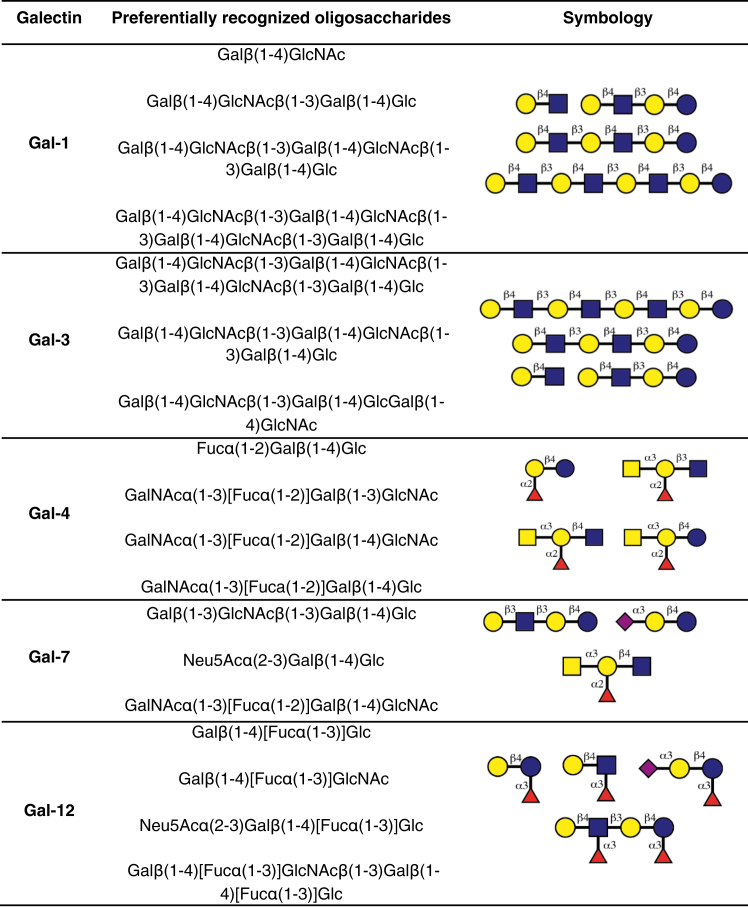
**Preferred ligands for individual galectins by SPA.** Glycans are depicted following the Symbol Nomenclature for Glycans (SNFG) (101). SPA, solid-phase assay.

The preferential HMO structures recognized by each individual galectin are summarized in Figure 3.

### ITC assays support preferential ligand-binding activity displayed by human galectins

ITC is generally regarded as the gold standard approach for determining affinities and thermodynamic parameters of protein−carbohydrate interactions. Thus, we validated interactions between human Gal-1, -3, -4, -7, and -12 and preferentially recognized glycan structures by ITC (Table 1, Fig. S2).

**Table 1:** ITC evaluation of thermodynamic binding parameters for human Gal-1, -3, -4, -7, and -12 toward the best three ligands previously identified by SPA

Galectin	Oligosaccharide	*K*_d_ (μM)	ΔH (kJ/mol)	-TΔS (kJ/mol)
Gal-1	LNnT	72 ± 8	−9.0 ± 0.9	−14.7 ± 0.8
	LNnH	69 ± 7	−11.8 ± 1	−11.9 ± 0.8
	LNnO	73 ± 7	−12.7 ± 2	−10.9 ± 0.0
Gal-3	LNnT	95 ± 9	ND∗	ND∗
	LNnH	42 ± 5	−6.2 ± 0.6	−19 ± 2
	LNnO	40 ± 3	−4.6 ± 0.4	−21 ± 2
Gal-4	2′-FL	130 ± 9	ND∗	ND∗
	BGA2	65 ± 5	−25 ± 2	1.1 ± 0.2
	BGA6	51 ± 3	−32 ± 3	7.4 ± 0.6
Gal-7	LNT	106 ± 8	ND∗	ND∗
	3′-SL	97 ± 9	ND∗	ND∗
	BGA2	103 ± 8	ND∗	ND∗
Gal-12	3-FL	56 ± 5	−66 ± 5	41 ± 3
	Le^X^	80 ± 9	ND∗	ND∗
	SFL	151 ± 11	ND∗	ND∗

No good fit of the thermodynamic parameters could be obtained for binding affinities above 80 μM (ND∗, not determined).

Results showed binding affinities comparable to those obtained by competitive SPA. Moreover, data analysis revealed that binding affinities were not too different among selected cases, with *K*_D_ values ranging between 40 and 151 μM, corresponding to relative ΔΔG values smaller than 1 kcal/mol (4 kJ/mol). Furthermore, for those cases in which the thermodynamic profiles could be determined with good accuracy, a typical enthalpy-entropy compensation was observed for the interaction of Gal-4 with BGA2 and BGA6 and of Gal-12 with 3-FL. In contrast, the interaction of Gal-3 with the tri- (LNnH) and tetra-LacNAc (LNnO) analogues was clearly favored by entropy, still displaying a favorable (small) enthalpy. A gain in entropy was also observed for the interactions of human Gal-1 with the di-, tri-, and tetra-LacNAc analogues, which showed very similar dissociation constants (Table 1, Fig. S2), consistent with those reported for Gal-1/LacNAc interactions (76 μM at 298 K) (59) and with the preferential recognition of the terminal LacNAc moiety (48) (see Discussion below). The observed stabilizing enthalpy should be mainly associated with the number of direct hydrogen bonds (HBs) established between the galectins and the glycosylated ligands (60), as well as with the key aromatic-sugar stacking CH-π interaction provided by the key Trp ubiquitous in all galectins and the nonpolar face of the β-Gal moiety (61). Nevertheless, and given the rather shallow architecture of galectin binding sites, water-mediated interactions before and after the formation of the complex may also play a key role, contributing to the enthalpy and entropy components (60). It has also been described that conformational entropy is also important for galectin binding, both from the ligand point of view, as for the interaction of the CRD of Gal-3 with the BGA2 and BGB2 antigens (47) and from the galectin’s side, as reported for the recognition of LacNAc by Gal-1 (62). In turn, human Gal-3 showed increased binding affinities for LNnT, LNnH, and LNnO (*K*_d_ values of 95, 42, and 40 μM, respectively), all presenting type II LacNAc subunits. As mentioned above, it has been described that Gal-3 exclusively binds to the internal Gal units in polyLacNAc moieties (52). Since the entropy term is fairly favorable for the longer LNnH and LNnO glycans, it is tempting to propose that statistical rebinding of the internal Gal rings provides additional impetus for the interaction. Moreover, HBs and Trp-Gal stacking interactions lead to a negative ΔH value, although the role of solvation cannot be discarded (61).

For human Gal-4, we were able to confirm the preferential and similar affinity toward blood group A tetrasaccharide antigens type 2 and 6 (*K*_d_ = 65 and 51 μM, respectively) than for the 2′-FL trisaccharide (*K*_d_ = 130 μM) (Table 1, Fig. S2). These results strongly suggest that the reducing end residue (Glc *versus* GlcNAc) does not significantly alter Gal-4 affinity, while substitution with α(1–3) GalNAc in the terminal galactose considerably improves Gal-4 interactions (56).

Prototype human Gal-7 exhibited a broad spectrum of glycan-binding preferences, including neutral, fucosylated, and sialylated HMOs, as shown by the very similar binding affinities of this lectin with LNT (as previously reported) (41), 3′-SL, and BGA2 (*K*_d_ values of 106, 97 and 103 μM, respectively, Table 1, Fig. S2).

Finally, the interaction of human full-length Gal-12 with 3-fucosylated glycans (3-FL, Le^X^, and SFL) showed similar *K*_d_ values to murine Gal-12 (14), with 3-FL as the best ligand (Table 1, Fig. S2). Replacement of Glc in 3-FL by GlcNAc (resulting in Le^X^) decreased affinity, while 3′-sialylation impaired interactions with this lectin. These results indicate that, in contrast to other members of the galectin family, human Gal-12 has unique binding preferences toward 3-fucosylated oligosaccharides.

### Toward the 3D structure of the galectin-glycan complexes through computational studies

**Figure 4:**
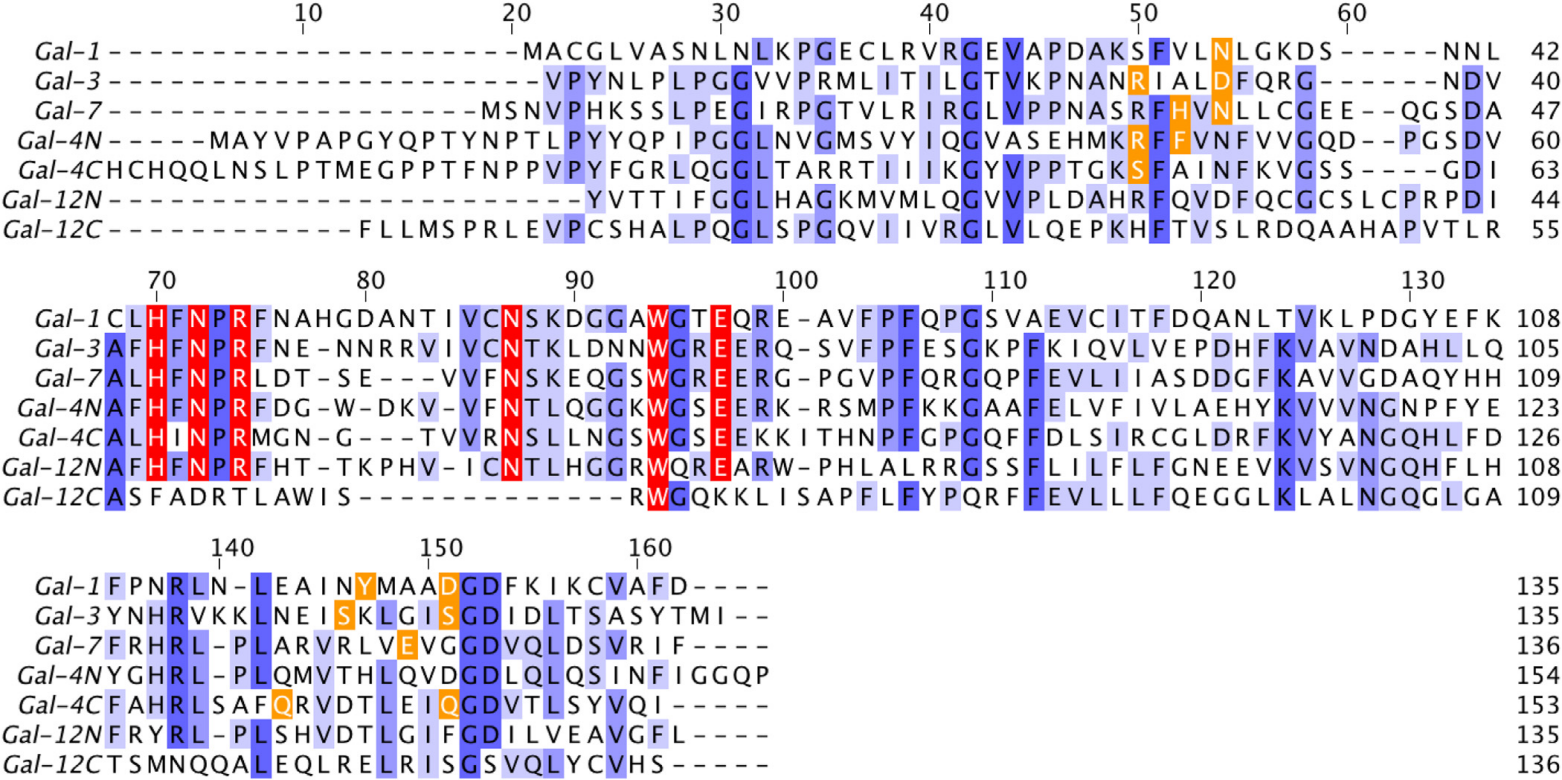
**Sequence alignment of human galectin-1, -3, -7, -4N, -4C, -12N, and -12C.** Sequences were aligned using ClustalW and visualized with Jalview (102). Each carbohydrate recognition domain (CRD) consists of 135 to 165 amino acids, arranged on two antiparallel β-sheets composed of six (F′-F5 and S1-S6) β-strands. Residues were colored by identity score ranging from *blue* (identical) to *white* (nonidentical). Highly conserved amino acid residues essential for recognition are highlighted in *red*, and specific residues relevant for recognition are marked in *orange*.

We next sought to characterize structural features of these interactions *in silico*, in an attempt to identify the key amino acid residues responsible for glycan recognition. Thus, we performed docking and molecular dynamics (MD) simulations with human Gal-1, -3, and -7 based on the reported crystal structures with preferentially recognized glycans (Table 1). Since no experimental structure of any full-length human tandem-repeat type galectin has been reported to date, the isolated N- and C-terminal CRDs of human Gal-4 and human Gal-12 were used for docking. In all cases, we selected the best docked conformations, and the generated complexes were submitted to 500 ns MD simulations. Sequences of evaluated galectins (or corresponding CRDs) are shown in Figure 4.

**Figure 5:**
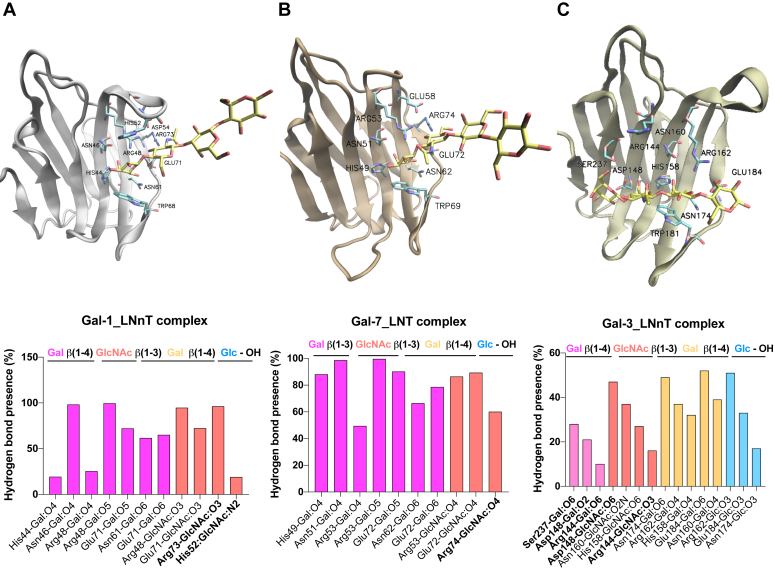
**MD studies of human Gal-1, Gal-3, and Gal-7 in complex with LNnT or LNT.** MD simulations of galectin-glycan complexes for (*A*) Gal-1-LNnT, (*B*) Gal-7-LNT, and (*C*) Gal-3-LNnT. Galectin CRDs are depicted in new *cartoon* representation, and ligands are depicted in *licorice*. Key hydrogen bond interactions for the galectin-oligosaccharide complexes obtained by MD simulations are analyzed. Bar graphs show the frequency of hydrogen bonds (HB) considering HB donors and HB acceptors separated by “-”. Nonconserved amino acid residues are shown in *bold*. CRD, carbohydrate recognition domain; MD, molecular dynamics; LNnT, lacto-N-neotetraose; LNT, lacto-*N*-tetraose.

First, we performed docking and MD studies of human Gal-1, Gal-3, and Gal-7 in complex with LNnT or LNT to dissect differences in glycan-binding preferences (Fig. 5). According to the preferential recognition of Gal-1 and Gal-7 toward terminal LacNAc residues on polylactosamine sequences (52), MD simulations of Gal-1-LNnT and Gal-7-LNT complexes showed the terminal galactose residue presenting CH-π stacking interactions with the conserved Trp68^hGal-1^/Trp69^hGal-7^, while the reducing-end lactose was disposed toward the solvent. In contrast, for the Gal-3-LNnT complex, the internal galactose was stacked with Trp181, consistent with the preferential recognition of Gal-3 for internal Lac/LacNAc moieties (52).

For Gal-1-LNnT and Gal-7-LNT complexes (Fig. 5, *A* and *B*), the LacNAc core at the nonreducing end was disposed toward the most conserved subsites C and D, adopting a similar conformation to that previously observed for LacNAc type II and type I in complex with Gal-1 and Gal-7, respectively. Residues His44^hGal-1^/His49^hGal-7^, Asn46^hGal-1^/Asn51^hGal-7^, Arg48^hGal-1^/Arg53^hGal-7^, Asn61^hGal-1^/Asn62^hGal-7^, and Glu71^hGal-1^/Glu72^hGal-7^ are involved in key HBs, while Trp68^hGal-1^/Trp69^hGal-7^ in CH-π interactions, as observed at the Gal-1-LacNAc (II) and Gal-7-LacNAc (I) crystal structures (54, 59, 62, 63) (Fig. 5, *A* and *B*). In addition, for the Gal-1-LNnT complex, HB interactions between Arg73 and GlcNAc HO-3 and His52 and GlcNAc HN-2 were observed (Fig. 5*A*), while a HB interaction between Arg74 and GlcNAc HO-4 was detected for the Gal-7-LNT complex (Fig. 5*B*).

In contrast, in the Gal-3-LNnT complex, the internal galactose residue was stacked with the conserved Trp181. MD studies showed key HBs of the LNnT reducing-end lactose core with conserved residues His158, Asn160, Arg162, Asn174, Trp181, and Glu184, and CH-π interactions with Trp181 (Fig. 5*C*). In addition, favorable contacts were observed with the terminal LacNAc unit, including those between terminal Gal and Ser237, Asp148, and Arg144, and those involving the GlcNAc and Asp148, Asn160, His158, and Arg144 (Fig. 5*C*).

**Figure 6:**
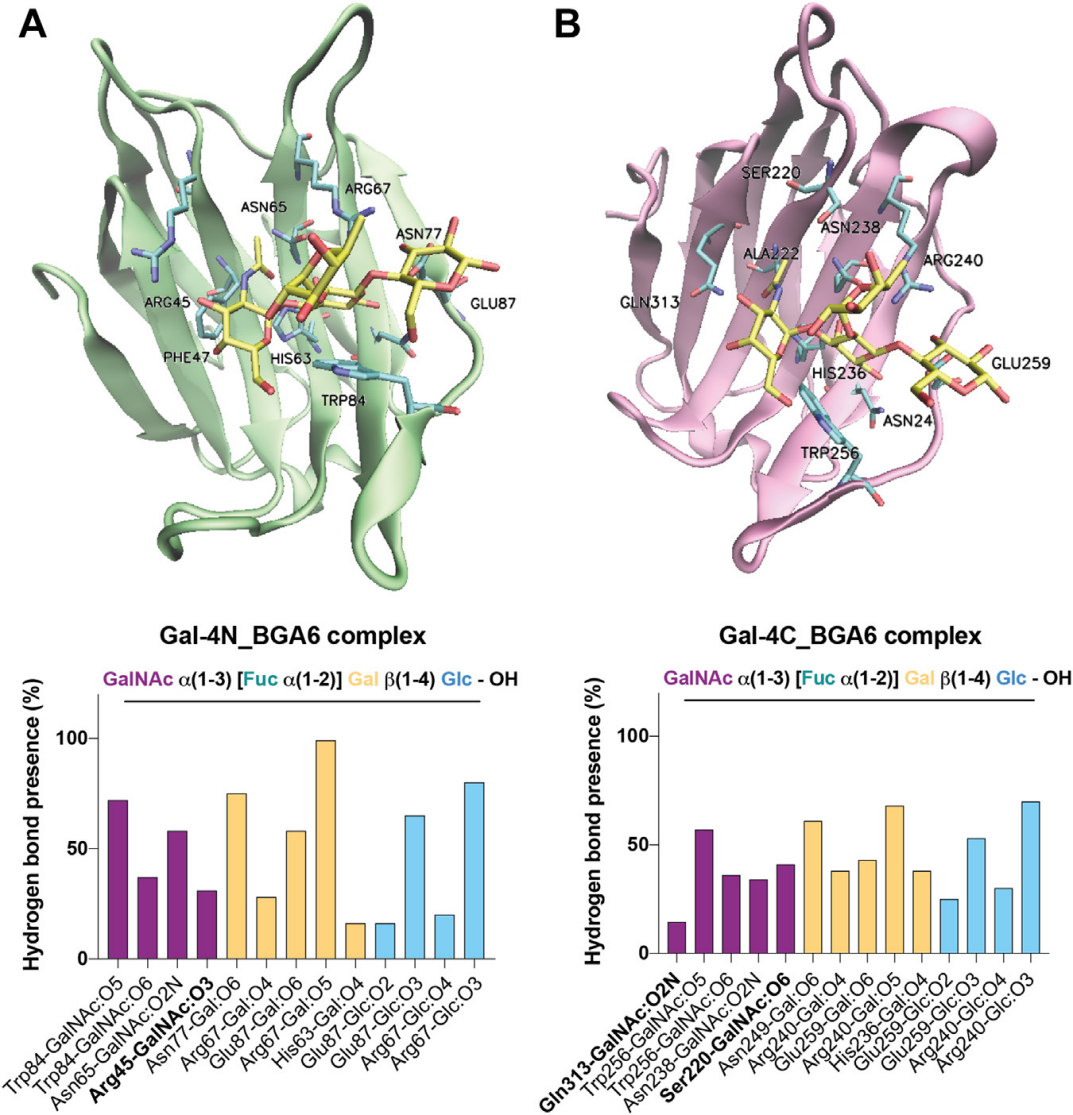
**MD studies of Gal-4 N-and C-terminal CRDs in complex with BGA6.** MD simulations of (*A*) Gal-4N-BGA6 and (*B*) Gal4C-BGA6 complexes. Gal-4 CRDs are depicted in new *cartoon* representation. BGA6 is depicted in *licorice*. Key HB interactions for the Gal-4N- and Gal4-C-BGA6 complexes (*A* and *B*, respectively) were obtained by MD simulations. Bar graphs show frequency of hydrogen bonds considering hydrogen bond donors and hydrogen bond acceptors separated by “-”. Nonconserved amino acid residues are shown in *bold*. BGA6, blood group A antigen tetraose 6; CRD, carbohydrate recognition domain; HB, hydrogen bond; MD, molecular dynamics.

Next, we evaluated both human Gal-4 CRDs in complex with the preferred full-length human Gal-4 glycan ligand, BGA6 (Fig. 6). In the MD simulation, HB interactions were predicted for the lactose core of BGA6 and conserved Gal4-N amino acid residues including His63, Arg67, Asn77, and Glu87, and CH-π interactions with Trp84 (Fig. 6*A*). Notably, no HBs were present in the simulation for any of the fucose OH groups, as this residue faced the bulk solvent (55). Additional favorable interactions were observed between αGalNAc residue and amino acid residues Arg45, Asn65, and Trp84, in full agreement with the proposed interactions for the Gal-4N-BGA6 complex based on NMR data (55), where HO-2 and HO-3 at the reducing-end Glc ring were facing the lectin, and a favorable HB interaction was proposed between HO-2 at the reducing-end Glc moiety and Glu87 (Fig. 6*A*). These GalNAc-Gal-4N interactions may contribute to the preferential affinity of Gal-4 toward BGA6 when compared to 2′-FL.

The Gal-4C-BGA6 MD simulations showed conserved HBs between the type II lactose core of BGA6 and Gal-4C amino acid residues His236, Asn238, Arg240, Asn249, Trp256, and Glu259, and CH-π stacking with Trp256 (Fig. 6*B*). Similarly to the Gal-4N-BGA6 complex described above, no HBs were observed for the fucose HO groups, with this residue facing the solvent. Additionally, the terminal GalNAc moiety presented several HBs including not only those with Trp256, Asn238, and Ser220 previously described (64). These GalNAc-Gal-4C interactions were further validated by STD-NMR analysis, as described in the accompanying paper (56).

**Figure 7:**
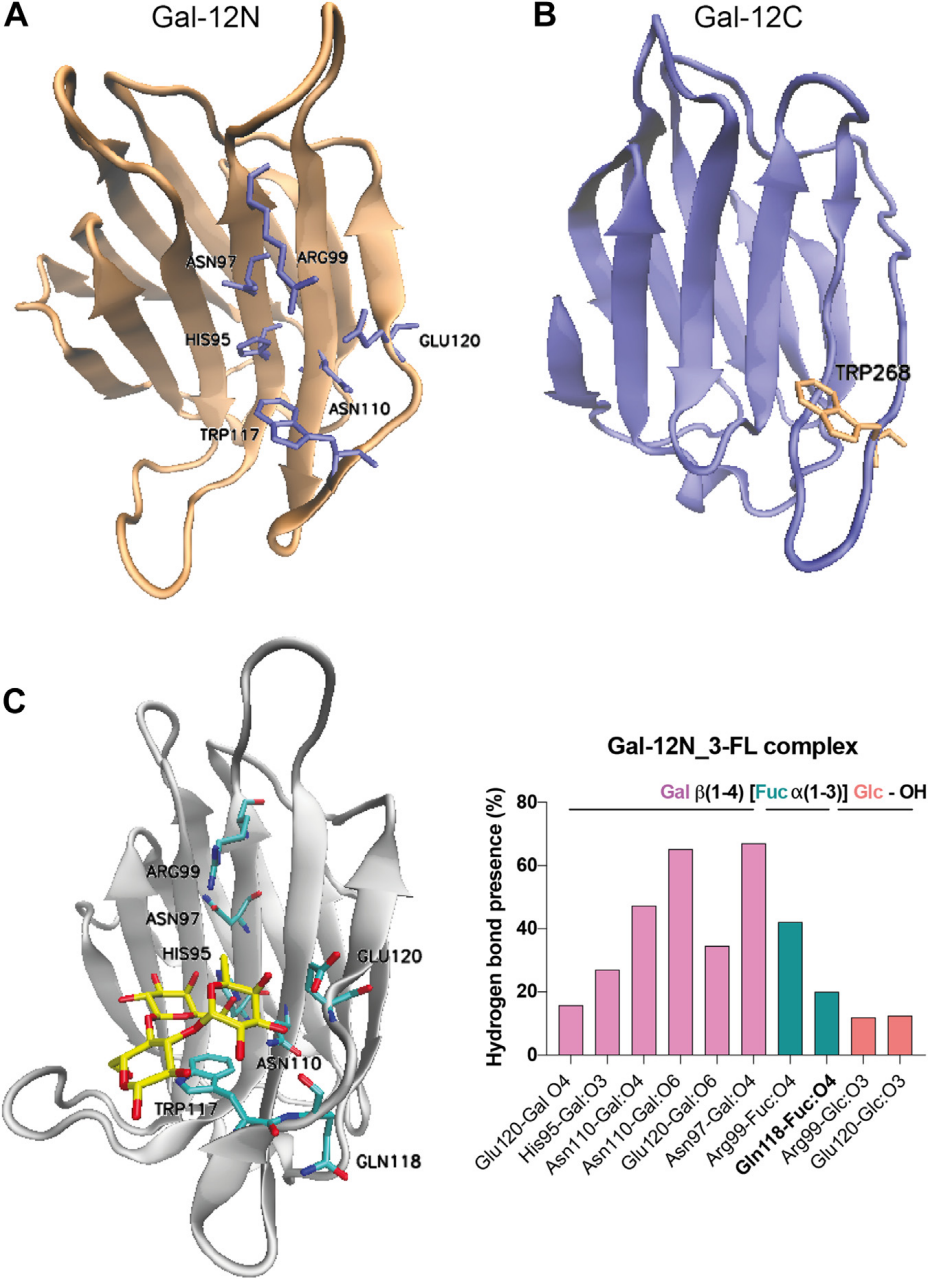
**Computational studies for Gal-12-3-FL complexes.***A* and *B*, homology model structures for the (*A*) N-terminal and (*B*) C-terminal domains of human Gal-12. Gal-12 CRDs are presented in new *cartoon* representation and essential conserved residues from the ligand binding groove are shown in *licorice*. *C*, MD simulations of the Gal-12N-3-FL complex. Gal-12 CRDs are depicted in new *cartoon* representation, and 3-FL in licorice. Key HB interactions were obtained by MD simulations. Bar graphs show frequency of hydrogen bonds considering HB donors and HB acceptors separated by “-”. Nonconserved amino acid residues are shown in *bold*. 3-FL, 3-fucosyllactose; HB, hydrogen bond; CRD, carbohydrate recognition domain; MD, molecular dynamics.

From a structural perspective, Gal-12 exhibits two highly distinct CRDs: while its N-terminal CRD exhibits significant homology to those in other galectin members, its C-terminal domain differs from the family consensus sequence, only presenting the highly conserved Trp268 residue responsible for stacking interactions with the hydrophobic face of galactose (Fig. 4) (65). Given that no experimental structure of Gal-12 is yet available, we generated human Gal-12 N- and C-terminal domain structural models by comparative modeling based on the structure of those in tandem-repeat galectins (Gal-4, Gal-8, and Gal-9) (Fig. 7, *A* and *B*). All models were characterized, presenting a GA341 score of 1.00, and the best ones were selected based on the final molpdf scores. Next, considering our previous results on full-length human Gal-12-glycan binding affinities, we used Gal-12N and Gal-12C structural homology models and conducted MD simulations for Gal-12N-3-FL and Gal-12C-3-FL complexes. While the human Gal-12C CRD complexed with 3-FL was not stable, the human Gal-12N-3-FL complex showed good stability and tight HB stabilizing interactions (Fig. 7*C*). Indeed, the core lactose from 3-FL presents conserved HBs with His95, Asn97, Arg99, Asn110, and Glu120, and CH-π interactions with Trp117 (Fig. 7*C*). Furthermore, the fucose residue, albeit disposed toward the solvent, still displayed HB interactions with Arg99 (one of the highly conserved residues responsible for galectin recognition of Lac/LacNAc derivatives).

### ^1^H-STD-NMR studies for Gal-4 selective ligands

**Figure 8:**
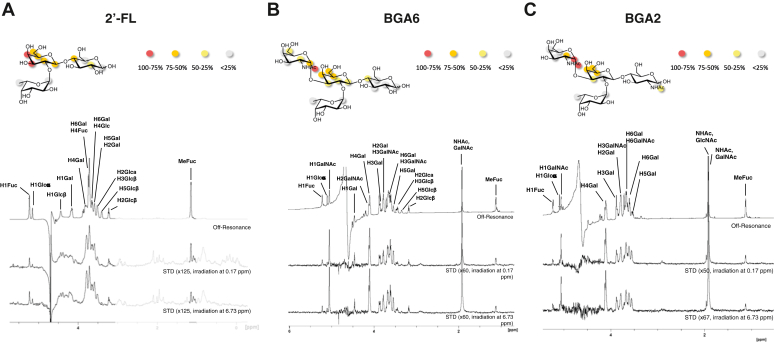
**1H-STD-NMR studies for Gal-4 in complex with 2'-FL, BGA6 and BGA2.** NMR analysis for Gal-4-glycan complexes with (*A*) 2′-FL, (*B*) BGA6 and (*C*) BGA2. (*Upper* panels). Epitope mapping for each glycan determined by 1H-STD NMR experiments (based on irradiation at 0.17 ppm). *Circles* indicate the positions whose STD intensity is reported (normalized against the most intense STD), and are color coded according to this normalized STD intensity. (*Lower* panels) ^1^H-STD-NMR spectra of a 2.5 mM glycan solution in the presence of 50 μM Gal-4 (ratio 50:1). First spectrum: reference spectrum with annotation for those signals showing STD intensity; second spectrum: STD spectrum with irradiation of the protein on the aliphatic region (0.17 ppm); third spectrum: STD spectrum with irradiation of the protein on the aromatic region (6.73 ppm). BGA6, blood group A antigen tetraose 6; STD, saturation transfer difference.

Given the peculiar glycan-binding activity of Gal-4 and its less explored functional activities, we further studied the binding of full-length human Gal-4 to 2′-FL, BGA2, and BGA6 glycans by ligand-based NMR experiments. Detailed information on the binding epitope was obtained by ^1^H-STD-NMR (Fig. 8), after ^1^H (and ^13^C) resonances of all studied compounds were assigned through standard TOCSY, NOESY, and heteronuclear single quantum coherence (HSQC) experiments. Annotated HSQC spectra and tables containing ^1^H and ^13^C chemical shifts have been included in the Supplementary material (Tables S2–S4, Figs. S3–S5).

For all three ligands analyzed, the central β-Gal residue constitutes the main Gal-4 binding epitope, while the additional α-GalNAc residue present in BGA6 and BGA2, and in particular H1, H2, and the NHAc methyl are also facing the protein surface. In contrast, and in accordance with the MD simulations described above, the protons of the fucose moiety did not show any significant STD intensities, assessing that this residue was not part of the binding epitope for any of the ligands tested.

### BGA6 inhibits Gal-4-driven IL-6 secretion by human activated PBMCs

Seeking for selective glycan inhibitors that could target the proinflammatory activity of Gal-4 (66), we further studied the ability of BGA6 to interrupt Gal-4-driven production of IL-6, a central proinflammatory cytokine implicated in autoimmunity, infection, and cancer (67).

**Figure 9:**
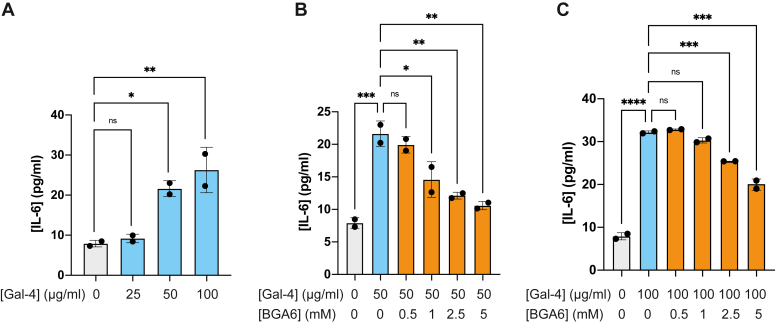
**BGA6 prevents Gal-4-driven IL-6 production by human activated peripheral blood mononuclear cells (PBMCs).***A*, IL-6 secretion by human PBMCs exposed to anti-CD3/CD28 mAbs to activate the T cell population and cultured with human recombinant Gal-4. *B* and *C*, human IL-6 ELISA determination on supernatants from PBMCs treated with (*B*) 50 μg/ml or (*C*) 100 μg/ml of Gal-4 and increasing concentrations of BGA6. One-way ANOVA followed by Tukey’s posttest. Individual points are independent biological replicates. Data are presented as means ± S.D. and are from a representative of three independent experiments. ∗*p*< 0.05, ∗∗*p*< 0.01, ∗∗∗*p*< 0.001, and ∗∗∗∗*p*< 0.0001. BGA6, blood group A antigen tetraose 6; IL, interleukin.

Interestingly, Gal-4 induced a significant and dose-dependent increase in secretion of IL-6 by human-activated T lymphocytes in PBMCs (Fig. 9*A*). Notably, Gal-4-driven IL-6 secretion was effectively suppressed by BGA6 in a dose-dependent manner at concentrations of 50 μg/ml and 100 μg/ml (Fig. 9, *B* and *C*, respectively). These findings highlight BGA6 as a potent inhibitor of Gal-4 proinflammatory activity, with critical implications in a broad range of immune-mediated disorders.

## Discussion

Galectins have emerged as relevant therapeutic targets for inflammatory diseases, fibrosis, and cancer (3, 12), highlighting the potential of selective inhibitors targeting individual members of this family for treating these pathologic conditions. Although these proteins have been originally defined based on their affinity for β-galactosides, recent studies have shown distinct glycan-binding preferences for each particular galectin as well as for individual galectin CRDs (9, 14, 15, 41, 49, 55, 56, 68). With different technical approaches used to study lectin-glycan interactions, glycan microarrays have become the dominant technology (41). Screening by shotgun glycan microarray benefits from the high-throughput performance of these technologies, but glycan immobilization may affect lectin recognition (57, 58), as demonstrated using different technologies including ESI-MS and ITC (42). In this work, we simultaneously analyzed and compared the affinities of 21 glycans including HMOs and BGAs toward human Gal-1, -3, -4, -7, and -12, and identified unique molecular determinants implicated in their recognition profiles, providing new insights into galectin-glycan interactions.

By competitive SPA, we validated the previously described selectivity of human Gal-1, -3, and -7 using other experimental approaches (41, 42, 48, 54), including impaired binding to 6′-sialylated and 3-fucosylated lactose derivatives (42, 48, 68). Results confirmed the preference of human Gal-1 for neutral HMOs bearing nonreducing type II LacNAc, while human Gal-7 preferentially bound to type I LacNAc. In this sense, Hsieh *et al.*, described unique differences for the salt bridges formed by Glu58 on Gal-7 compared to those established by Asp54 on Gal-1 and Glu165 on Gal-3 (54). Additionally, Collins *et al.*, compared the crystallized structures of Gal-3 complexed with LNnT and LNT, and concluded that in the former, the binding conformation of the terminal β(1–4)-linked Gal moiety forces the terminal galactose to be much closer to the protein surface compared to that in LNT, thus favoring its interactions (69). In our MD analysis, the nonreducing end LacNAc on both Gal-1-LNnT and Gal-7-LNT tetrasaccharides adopted similar conformations than those shown in Gal-1-LacNAc (II) and Gal-7-LacNAc (I) complexes. For Gal-3-LNnT, the internal lactose core was disposed on the conserved subsites C and D on the Gal-3 CRD, presenting similar interactions to those reported for the Gal-3-LacNAc crystal structure (69), and consistent with the preferential recognition of Gal-3 toward internal LacNAc units (52). In contrast to human Gal-3, no substantial gain in human Gal-1 affinity was observed for higher HMOs such as LNnH and LNnO, compared to LNnT; these differences have also been observed by ESI-MS (42) and frontal affinity chromatography (48), and attributed to the capacity of human Gal-3 (but not Gal-1) to bind internal LacNAc units. With no currently available experimental structures of galectins with polyLacNAc oligosaccharides like LNnH and LNnO, further studies are needed in order to understand the atomistic determinants implicated in these interactions.

From a biological perspective, HMO composition is influenced by several factors, including fucosyltransferase 2 (FUT2) genotype. *FUT2*^*−/−*^ mothers do not produce α(1–2)-fucosylated HMOs, such as 2′-FL, and women with the Le locus produce the highest amounts of 3-FL (70). Moreover, concentration of HMOs vary with time, as previous studies in the first 24 months of lactation showed that the majority of HMO concentrations decrease significantly, while specific structures remain unchanged (*e.g.*, 2′-FL), or increased (*e.g.*, 3′-SL, 3-FL) (71).

Given that cow´s milk exhibit lower oligosaccharide concentrations than human milk, and present different composition, both in specific concentration and structure of certain oligosaccharides particularly sialylated and fucosylated structures (72), infant formulae have recently been supplemented with specific HMOs. Particularly, in clinical studies no adverse effects were reported for 2′-FL. Moreover, studies of milk supplementation with this trisaccharide for infants resulted in increased number of beneficial bacteria and decrease number of pathogenic bacteria (73), leading to formulation of 2′-FL-supplemented infant formulas. Lacto-*N*-neotetraose (LNnT), DFL, LNT, and 3′-SL sodium salt have also received market authorization as new food ingredients in the United Kingdom, the United States, the European Union, Russia, Israel, and Singapore. Furthermore, 3-FL has been recently approved in the US and Australia.

The immunomodulatory roles of HMOs have been widely described showing diverse beneficial effects (18, 19). In fact, these oligosaccharides behave as soluble analogues of glycostructures present on the epithelial cell surface and glycocalyx, thus allowing competition for microbiota (35), and being fermented by bacteria (50). Further, beyond their specific functions in the gut and considering that approximately 1% of ingested HMOs are absorbed, their roles as systemic immunomodulators are also under evaluation.

Given that Gal-4 is preferentially expressed by epithelial cells of the intestinal tract and secreted to the extracellular milieu (74), our results demonstrating its affinity for 2′-FL lead to the hypothesis that part of the beneficial roles of this trisaccharide could be mediated by interactions with this lectin. Moreover, a recent study proposed that 2′-FL can modulate immune responses against enteric viruses, with results showing increased levels of Gal-4 expression in cocultures of intestinal epithelial cells and monocyte-derived dendritic cells stimulated in the presence of this oligosaccharide (75).

In turn, human Gal-12 showed a unique recognition pattern for 3-fucosylated structures, consistent with previous glycan microarray data for human Gal-12N CRD (https://www.functionalglycomics.org/glycan-array/1003213) and our results obtained for murine Gal-12 (14). This specificity resembles glycan-binding preferences of C-type lectins such as DC-SIGN (76), more than those displayed by galectins. Our MD studies suggested that recognition of 3-FL was essentially mediated by the human Gal-12 N-CRD (Fig. 7), which is in accordance with the lack of conserved amino acid residues on its C-CRD, apart from the highly conserved Trp268 residue (Fig. 4). The role of human Gal-12 C-CRD still remains unclear, and future studies focused on glycolipids as ligands should be performed, considering the hydrophobicity of this galectin, its preferential localization within adipose tissue and its compartmentalization in lipid droplets (14, 77, 78). Docking studies showed that the fucose residue in 3-FL exhibit favorable interactions with Gln118, a residue also present in the murine Gal-12 ortholog (14) and unique for Gal-12, replacing the glycine residue present on other galectins including tandem-repeat Gal-4, -8, and -9, and a lysine residue on Gal-10 (Fig. 4). These results suggest that Gln118 may be responsible for these unique Gal-12 binding preferences. Albeit the function of this lectin in inflammatory and intestinal pathologies still remains uncertain, future work is needed to unveil a potential role of this protein in the immunomodulatory properties of 3-FL.

It has been hypothesized that the interaction of BGAs with human Gal-3, -4, and -7 might be associated with an evolutionary conserved role of these galectins, bridging a gap between innate and adaptive immunity (79). Since blood group A, B, or AB positive individuals cannot make the corresponding antiblood group antibodies, and microorganisms decorate themselves with BGAs as a molecular mimicry strategy for infection, it has been proposed that galectin recognition of AB0 BGAs may confer protection against these pathogens (44). In this sense, our results highlighting Gal-3 binding toward blood group A and B antigens with moderate affinity are in line with previous data (46, 68). The recognition of BGAs by human Gal-7 (49) and full-length human Gal-4 and its isolated CRDs (41, 55, 56) were also validated in this study. Since Gal-7 is preferentially expressed in skin, and Gal-4 is abundant in the gut, these interactions could be associated with immune defense mechanisms in response to molecular mimicry of pathogens (44, 79). These effects may not be restricted to human Gal-3, -4, and -7, since similar results have been documented for Gal-8 (80) as well as full-length Gal-9 and its CRDs (81).

Although blood group A glycans are recognized by different galectin family members, an important selectivity was observed for full-length human Gal-4. Our *in silico* simulations were consistent with STD-NMR experiments, showing no relevant interactions with the Fuc residue and a critical role of αGalNAc on glycan recognition. The affinity of Gal-4 toward GalNAcα(1–3)Galβ(1–4)Glc/GlcNAc trisaccharide has not been evaluated herein, since biosynthetically 2′-fucosylation precedes addition of the α(1–3)GalNAc terminal residue (82). Our findings *in vitro* show that BGA6 inhibits Gal-4-dependent IL-6 secretion in a dose-dependent fashion, highlighting the potential role of this natural oligosaccharide as a Gal-4 inhibitor. Given the critical roles of Gal-4 in cell adhesion and wound healing, intestinal inflammation, and tumor progression (74), further exploration of the inhibitory capacity of BGA6 in Gal-4-driven inflammation *in vivo* is warranted.

In conclusion, our study supports the idea that HMOs could act as “stripping agents” for galectins, as previously proposed (83). Considering the high concentration of oligosaccharides in human milk, their galectin-dependent inhibitory capacity could be associated to specific roles in the gastrointestinal tract or general roles in modulating systemic immunity (83). In turn, and similar to the Siglec-sialome axis (84), galectin-BGA interactions may play key roles in the evolutionary arms-race between mammals and their pathogenic microbes.

## Experimental procedures

### Reagents

Lactose was purchased from Sigma-Aldrich, while all other oligosaccharides evaluated in this study were purchased from Elicityl.

### Recombinant expression and purification of galectins

Human Gal-1, -3, and -7 were recombinantly expressed and purified by affinity chromatography as previously described (85, 86, 87). Briefly, Gal-1, -3, and -7 were produced in *Escherichia coli* BL21 (DE3) cells transformed with corresponding constructs based on vector pET22b (Novagen) and their expression was induced by the addition of 1 mM isopropyl-β-D-thiogalactoside once the absorbance (OD_600_) reached 0.5 during 16 h at 30 °C. Bacterial cultures were centrifuged (6000*g*, 30 min, 4 °C), pellet was suspended in buffer A (PBS with 4 mM β-mercaptoethanol) supplemented with 25 μM tosyl-L-lysine chloromethyl ketone (TLCK) and 0.5 mM PMSF, disrupted by sonication and centrifuged at 4 °C (16,000*g* for 30 min). Galectins were purified from supernatants by affinity chromatography on a lactosyl-Sepharose column using a 0.1 M lactose solution in buffer A for protein elution. Lectin-containing fractions were dialyzed against buffer A to remove lactose and stored at −20 °C until activity evaluation.

Recombinant human Gal-4 was cloned into the expression vector pET-28a-SUMO, designed to produce an N-terminal His-tagged SUMO fusion protein in which the tag could be cleaved using ubiquitin-like-specific protease 1 (ULP1; Sigma-Aldrich). Transformed *E. coli* BL21 (DE3) cells were cultured in LB media as previously described. Then, bacterial pellet was suspended in buffer B (10 mM Tris–HCl pH 7.5, 0.5 M NaCl supplemented with 14 mM β-mercaptoethanol, 25 μM TLCK, and 0.5 mM PMSF) with 20 mM imidazole and disrupted by sonication. The lysate was supplemented with Triton X-100 (1%) for protein stabilization, incubated for 30 min at 4 °C and centrifuged at 4 °C (16,000*g* for 30 min), before Gal-4 purification by Ni-NTA column preequilibrated with buffer B with 20 mM imidazole. The His6-SUMO-Gal-4 fusion protein was eluted at 250 mM imidazole in a step gradient. Protein fractions were concentrated using a 10 kDa cutoff centrifugal filter unit Amicon Ultra-15 (Millipore) and dialyzed against buffer B. The His6-tagged SUMO was cleaved by ULP1 protease for 16 h at 4 to 8 °C or 1 h at 30 °C. The sample was subsequently loaded onto a Ni-NTA resin column where Gal-4 was separated from ULP1 and His_6_-SUMO through elution with buffer B supplemented with 20 mM imidazole. Gal-4 was finally dialyzed against buffer A for subsequent experiments and stored at 4 to 8 °C until activity evaluation.

Human Gal-12 recombinant expression and purification was adapted from that previously described for murine full-length Gal-12 (14). In this case, the construct containing recombinant human Gal-12 was cloned into vector pET-11a. Briefly, the plasmid was cloned in *E. coli* Rosetta 2 (DE3) cells and cultured in LB medium as described previously and induced with 0.5 mM IPTG for 6 h. Bacterial pellets were resuspended in buffer C (100 mM Hepes, 50 mM NaCl pH 6.1 containing 8 mM β-mercaptoethanol) supplemented with 0.5% DOC, lysozyme (4 mg/ml), DNAse 0.5 U/ml (benzonase endonuclease), and protease inhibitors, 25 mM TLCK and 0.5 mM PMSF. Soluble fraction was loaded to a Q-Sepharose column (GE HealthCare), equilibrated in buffer C. Fractions containing protein were purified by a carboxymethyl Sepharose (CM Sepharose, GE HealthCare Life Sciences) cationic exchange resin and eluted increasing NaCl concentration. Gal-12 was then concentrated and dialyzed against buffer C using a Vivaspin 20 (30 kDa cutoff centrifugal filter, Sartorius).

Protein concentration during purification was determined by standard BCA Protein Assay (Thermo Fisher Scientific) and NanoDrop 2000 UV-Vis spectrophotometer quantification (Thermo Fisher Scientific). Protein purity was checked by 12% SDS-PAGE. Endotoxin-free recombinant galectins were obtained by purification on a Detoxi-Gel column (Thermo Fisher Scientific). Recombinant galectins were then sterilized using a 0.22 μm syringe filter and adjusted to 1 to 5 mg/ml in buffer A.

### Competitive solid-phase assays

The assay was adapted from Rapoport *et al.* (2010) (88). Briefly, 96-well plates (flat-bottom) were coated with 10 μg/ml asialofetuin (ASF, Sigma-Aldrich) in sodium carbonate buffer (pH 9.6) and incubated overnight at 4 °C. Then, wells were washed three times with 100 μl/well PBS-Tween 0.05% (w/v) and blocked with 100 μl/well PBS-bovine serum albumin (BSA) 2% (1 h, room temperature (RT)) in a humid chamber. Meanwhile, equal volumes of human recombinant Gal-1, -3, -4, -7, and -12 (20–40 μg/ml) in PBS-BSA 0.3% buffer were preincubated with the corresponding inhibitors in serial dilutions (2 h, 37 °C). After washing, galectins were detected using the corresponding antigalectin primary detection antibodies in PBS-BSA 0.3% w/v (1 h at RT). For Gal-1, wells were incubated with an in-house purified 100 ng/ml biotinylated rabbit anti–human Gal-1 polyclonal immunoglobulin G. For Gal-3, -4, and -7, a 1:500 dilution of the corresponding biotinylated anti-human galectin antibodies was used (BAF1154, BAF1227, and MAB13391, respectively, R&D Systems). For Gal-12, a rabbit anti-human Gal-12 polyclonal antibody (Santa Cruz Biotechnology, 1:700) was used. For Gal-1, -3, -4, and -7, plates were rinsed three times before adding 0.33 μg/ml horseradish peroxidase (HRP)-labeled streptavidin (Sigma-Aldrich) for 30 min. For Gal-12, an anti-rabbit secondary antibody conjugate coupled to HRP was incubated for 1 h at RT in PBS-BSA 0.3% w/v. After washing, 100 μl TMB solution (0.1 mg/ml tetramethylbenzidine and 0.06% H_2_O_2_ in citrate-phosphate buffer, pH 5.0) was added to plates. The reaction was stopped by adding a 2N H_2_SO_4_ solution. Absorbance was determined at 450 nm in a Multiskan MS microplate reader (Thermo Fisher Scientific).

### Isothermal titration calorimetry

All ITC experiments were performed by using a NanoITC (TA Instruments) under previously optimized conditions (89). A typical titration involved 20 injections at 300 s intervals of 2.5 μl aliquots of a 2.5 to 10 mM ligand solution into the sample cell (volume 200 μl) containing the different galectins (30–100 μM). The solutions were prepared by dissolving the glycan ligands in degassed PBS buffer at 298 K. The titration cell was continuously stirred at 300 rev/min. The heats of dilution of the ligands in the buffer were subtracted from the titration data. Due to the relatively low affinity of human galectins for monovalent glycans (>50 μM), the “low c-value” method was used for ITC measurements with the stoichiometry (*n*) fixed to 1.00 (90). Fitting was performed using the Nano Analyze software (TA Instruments; https://www.tainstruments.com/itcrun-dscrun-nanoanalyze-software/) to determine association constants (*K*_a_) and the enthalpy change (ΔH). No reliable fit of the thermodynamic parameters could be obtained for binding affinities above 80 μM.

### Ligand docking calculations

The structure of the publicly available Protein Data Bank (PDB) structure of Gal-1 (PDB 1GZW), −3 (PDB 4LBN), -4N (PDB 5DUV), -4C (PDB 4YM3), and −7 (PDB ID 4GAL) were edited for docking calculations using AutoDock Tools 1.5.6 software (https://autodock.scripps.edu/): polar hydrogens and partial charges were added explicitly, whereas the program automatically added other hydrogen atoms.

No crystal structures for Gal-1-LNnT and Gal-7-LNT complexes have been reported, while PDB ID 4LBN accounts for Gal-3-LNnT crystal structure (69). Thus, the X-ray crystallographic structures of Gal-1 bound to lactose (PDB code 1GZW) (59), Gal-3 bound to LNnT (PDB code 4LBN) (69) and Gal-7 bound to lactose (PDB code 4GAL) (63) were used as a starting geometry for these three galectins. Given the preferential recognition of Gal-1 and Gal-7 toward terminal LacNAc residues on polylactosamine sequences (52), the MD simulations for the Gal-1-LNnT and Gal-7-LNT complexes were carried out with the terminal galactose residue presenting CH-π stacking interactions with the conserved Trp68^hGal-1^/Trp69^hGal-7^, while the reducing-end lactose was disposed toward the solvent. In contrast, for the Gal-3-LNnT complex, the internal galactose was stacked with Trp181, consistent with the preferential recognition of Gal-3 for internal Lac/LacNAc moieties (52).

For the MD simulation of Gal-4N and -4C complexed with BGA6, the X-ray crystallographic structure of Gal-4N (PDB code 5DUV) (91) and -4C (PDB code 4YM3) (64) bound to lactose were used as starting geometries, respectively.

For human Gal-12 N- and C-CRDs, molecular homology models were generated for this analysis as previously described for murine Gal-12 N- and C-CRDs (14). The construction for human Gal-12N was based on the templates from Gal-9N structures (ID: 2D6N; 2D6M; 2D6K; 2D6L; 2D6P) and Gal-8N structures (ID: 2DYC; 3I8T). For human Gal-12C, the following structures were used as templates: Gal-4C (ID: 1X50), Gal-8C (ID: 3OJB; 4GXL) and Gal-9C (ID: 3NV1; 3NV4). Comparative models were designed with Modeller software (https://salilab.org/modeller/) (92). Five structural models were generated for Gal-12N- and Gal-12C-CRDs, and selection of the best one was based on Molpdfs score (Modeller Objective Function). All structural data were obtained from the RCSB-PDB database (Research Collaboratory for Structural Bioinformatics—Protein Data Bank—http://www.rcsb.org/). The 3D carbohydrate structures of glycans were obtained with the GLYCAM software (https://glycam.org). The edited structure of galectins and galectins’ CRDs were used for the docking procedure with AutoDock Vina 1.2 software (https://vina.scripps.edu/) (93).

Next, the more stable conformers of glycan compounds were manually docked into the carbohydrate-binding sites of each specific galectin family member by superimposing the terminal Gal residue with that of the crystallographic coordinates. The docking protocol was initially set to rigid condition with a size of the dock grid of 20 × 20 × 20 Å, which encompasses the binding site for the carbohydrate ligands. Exhaustiveness was initially set to 10 with all other parameters set on default values, then was increased to 100 for final dockings. The top-ranked complexes, sorted by binding energy values, were visually inspected for good stereochemical geometry and docking and further used as starting conformations for MD studies. For visualization, docking poses generated by AutoDock Vina were directly loaded into PyMol (http://www.pymol.org) through PyMOLAutodock/Vina Plugin (94).

### MD studies

After defining the initial conformation by docking studies with selected ligands, 500 ns MD runs were conducted for galectin-glycan complexes. In all cases, galectin-ligand complexes were solvated with explicit three-site point charge modeled (TIP3P) water molecules in an octahedral box, localizing the box limits 10 Å away from the protein surface. MD simulations were performed at 1 atm and 300 K, maintained with the Berendsen barostat and thermostat (95, 96), using periodic boundary conditions and Ewald sums (grid spacing of 1 Å) for treating long-range electrostatic interactions with a 10 Å cutoff for computing direct interactions. The SHAKE algorithm was applied to all hydrogen-containing bonds, allowing employment of a 2 fs time step for the integration of Newton’s equations. Amber ff19SB and GLYCAM-06j_1 force field parameters were used for galectins and glycans, respectively (97, 98, 99). The equilibration protocol involved a minimization of the initial structure, followed by 400 ps constant volume MD run heating the system slowly to 300 K. Finally, a 0.8 ns MD run at constant pressure was performed to achieve proper density. MD runs of 500 ns for galectin complexes with HMOs were performed. Frames were saved at 1 ps intervals. MD results were visualized with VMD software 1.9.1 (https://www.ks.uiuc.edu/Research/vmd/) (100) and analyzed with the Amber20 package (https://ambermd.org/) (99).

### Nuclear magnetic resonance

Full-length human Gal-4 was dissolved in PBS (50 mM sodium phosphate, 150 mM NaCl, pH 7.4, with 1 mM DTT), either in D_2_O or 90:10 H_2_O:D_2_O depending on the NMR experiment. The pH was adjusted with the required amount of NaOH and HCl or NaOD and DCl.

### ^1^H-saturation transfer difference NMR

All ^1^H-STD NMR experiments were acquired on an 800 MHz Bruker instrument equipped with a cryoprobe. The samples were prepared in the corresponding deuterated buffer. ^1^H-STD NMR spectra were acquired with 1024 scans, 2s of saturation time using a train of 50 ms Gaussian-shaped pulses, and 3 s of relaxation delay. The spin-lock filter applied to remove the residual signals of the lectin was set at 40 ms. For Gal-4, the temperature was 298 K. In addition, 50 μM of the lectin with 50 equivalents of the ligand were used. The on-resonance frequencies were set at 7.19 ppm (aromatic region) and at 0.52 ppm (aliphatic region), while the off-resonance frequency was set at 100 ppm. The experiments with Gal-4 and 2′-FL, BGA6 and BGA2 were acquired at 298 K. The on-resonance frequency was set at 7.03 ppm, and the off-resonance, at 100 ppm. The experiments were performed using 25 μM of human Gal-4 with 50 equivalents of tested ligands. The ^1^H-NMR resonances of the compounds were assigned through standard TOCSY (60 and 90 ms mixing times), NOESY (200–500 ms mixing times), and HSQC experiments. Subsequently, 500 μl samples were prepared by dissolving the purified compound in phosphate buffered saline 1X pH 7.4 prepared in D_2_O.

### Human PBMC preparation and activation

PBMCs were isolated from anonymous healthy volunteer buffy coats (Fundación Hemocentro Blood Bank, Buenos Aires, Argentina), using Ficoll-Hypaque density gradient (Lymphoprep). The samples were then centrifuged at 1500 rpm for 25 min, and the interface corresponding to mononuclear cells was carefully removed and diluted with PBS supplemented with 2% fetal bovine serum. To reduce platelet contamination, three washing steps with PBS 2% fetal bovine serum were performed, and freshly isolated PBMCs were subsequently resuspended in complete RPMI medium. To obtain a bead to T-cell ratio of 1:1, approximately 16 × 10^4^ PBMCs were activated with 2 μl of Dynabeads Human T-Activator CD3/CD28 (Gibco). After 24 h stimulation at 37 °C and 5% CO_2_, cells were incubated with human recombinant Gal-4 (25, 50, or 100 μg/ml) in the absence or presence of BGA6 (0.5, 1, 2.5, and 5 mM) for an additional time period of 72 h.

### IL-6 ELISA

Human IL-6 was determined in supernatants from activated PBMCs exposed or not to Gal-4 in the absence or presence of BGA6 using specific ELISA kits (BD Biosciences), according to the manufacturer's instructions. Briefly, 96-well plates (Costar) were coated with capture antibody during 18 h at 4 °C. Plates were then washed three times (PBS pH 7.4; 0.01% Tween-20) and incubated with blocking buffer (PBS/10% fetal bovine serum) for 1 h at RT. Then, samples and controls were incubated for 2 h. After four washing steps, a detection solution containing biotinylated secondary antibody and streptavidin-HRP was added for 1 h. After washing, plates were incubated with TMB solution (3,3′,5,5′-tetramethylbencidine) and 0.03% H_2_O_2_ in phosphate-citrate buffer (0.1 M citric acid; 0.1 M Na_2_HPO_4_). Reaction was stopped with 2N H_2_SO_4_, and the absorbance was measured at 450 nm using a plate spectrophotometer (Multiskan).

### Graphical representation

Molecular modeling structures were represented using VMD 1.9.3 visualization software (https://www.ks.uiuc.edu/Research/vmd/).

### Statistical analysis

Statistical analysis was performed using GraphPad Prism 9.0 software (GraphPad; https://www.graphpad.com/). Student’s *t* test was used for unpaired data. Two-way ANOVA and Dunnett’s or Tukey post tests were used for multiple comparisons. *p* values of 0.05 or less were considered significant. Exact *p* values are reported in all figures.

## Data availability

The datasets used and/or analyzed during the current study are available from the corresponding authors on reasonable request.

## Supporting information

This article contains supporting information.

## Conflict of interest

The authors declare that they have no conflicts of interest with the content of this article.
