# Supplementary material for: Exploring galectin interactions with human milk oligosaccharides and blood group antigens identifies BGA6 as a functional galectin-4 ligand
Source: J Biol Chem. 2024 Jul 14;300(8):107573. doi: 10.1016/j.jbc.2024.107573 (PMC11367503; doi:10.1016/j.jbc.2024.107573)
Supplement: Supporting Information [file mmc1.docx]

***Supporting Information***

**Exploring Galectin Interactions with Human Milk Oligosaccharides and Blood Group Antigens Identifies BGA6 as a Functional Galectin-4 Ligand**

Alejandro J. Cagnoni^1,2,ξ^, Mora Massaro^1,ξ^, Anabela M. Cutine^1,2^, Ana Gimeno^3^, Juan M. Pérez-Sáez^2^, Montana N. Manselle Cocco^2^, Sebastián M. Maller^2^, Santiago Di Lella^4,5^, Jesús Jiménez-Barbero^3,6,7,8^, Ana Ardá^3,6^, Gabriel A. Rabinovich^2,5,*^, Karina V. Mariño^1,9,*^

^1^Laboratorio de Glicómica Funcional y Molecular, Programa de Glicoinmunología, Instituto de Biología y Medicina Experimental (IBYME), Consejo Nacional de Investigaciones Científicas y Técnicas (CONICET), C1428 Buenos Aires, Argentina.

^2^Laboratorio de Glicomedicina, Programa de Glicoinmunología, Instituto de Biología y Medicina Experimental (IBYME), Consejo Nacional de Investigaciones Científicas y Técnicas (CONICET), C1428 Buenos Aires, Argentina.

^3^ CIC bioGUNE, Bizkaia Technology Park, Building 800, 48160 Derio, Bizkaia, Spain.

^4^Instituto de Química Biológica, Ciencias Exactas y Naturales (IQUIBICEN-CONICET), C1428 Ciudad de Buenos Aires, Argentina.

^5^Departamento de Química Biológica, Facultad de Ciencias Exactas y Naturales, Universidad de Buenos Aires, C1428, Ciudad de Buenos Aires, Argentina.

^6^ Ikerbasque, Basque Foundation for Science, Maria Diaz de Haro 3, 48013 Bilbao, Bizkaia, Spain.

^7^ Department of Organic & Inorganic Chemistry, Faculty of Science and Technology University of the Basque Country, EHU-UPV, 48940 Leioa, Spain.

^8^ Centro de Investigación Biomédica En Red de Enfermedades Respiratorias, 28029 Madrid, Spain.

^9^ Universidad Argentina de la Empresa (UADE), Instituto de Tecnología (INTEC), C1073

Ciudad de Buenos Aires, Argentina.

* Correspondence to: G.A.R, gabyrabi@gmail.com; K.V.M., kmarino@ibyme.conicet.gov.ar.

^ξ^ These authors contributed equally to this work.

**Running title:** HMO and blood group antigens recognition by human galectins

**Contents**

**Figure S1.** Competitive solid phase assays for the evaluation of human galectins in interaction with HMOs and blood group glycans.

**Table S1.** IC_50_ values obtained by SPAs for human Gal-1, -3, -4, -7 and -12 with tested oligosaccharides.

**Figure S2.** Experimental calorimetric data by isothermal titration at 298 K.

**Table S2.** ^1^H and ^13^C chemical shifts (ppm) of 2’-fucosyllactose at 298K.

**Figure S3.** ^1^H,^13^C-HSQC spectrum of 2’-fucosyllactose recorded at 298K. Resonance assignments have been annotated.

**Table S3.** ^1^H and ^13^C chemical shifts of A type 2 antigen (BGA2) at 298K.

**Figure S4.** ^1^H,^13^C-HSQC spectrum of BGA2 recorded at 298K. Resonance assignments have been annotated.

**Table S4.** ^1^H and ^13^C chemical shifts of A type 6 antigen (BGA6) at 298K.

**Figure S5.** ^1^H,^13^C-HSQC spectrum of BGA6 recorded at 298K. Resonance assignments have been annotated.

**
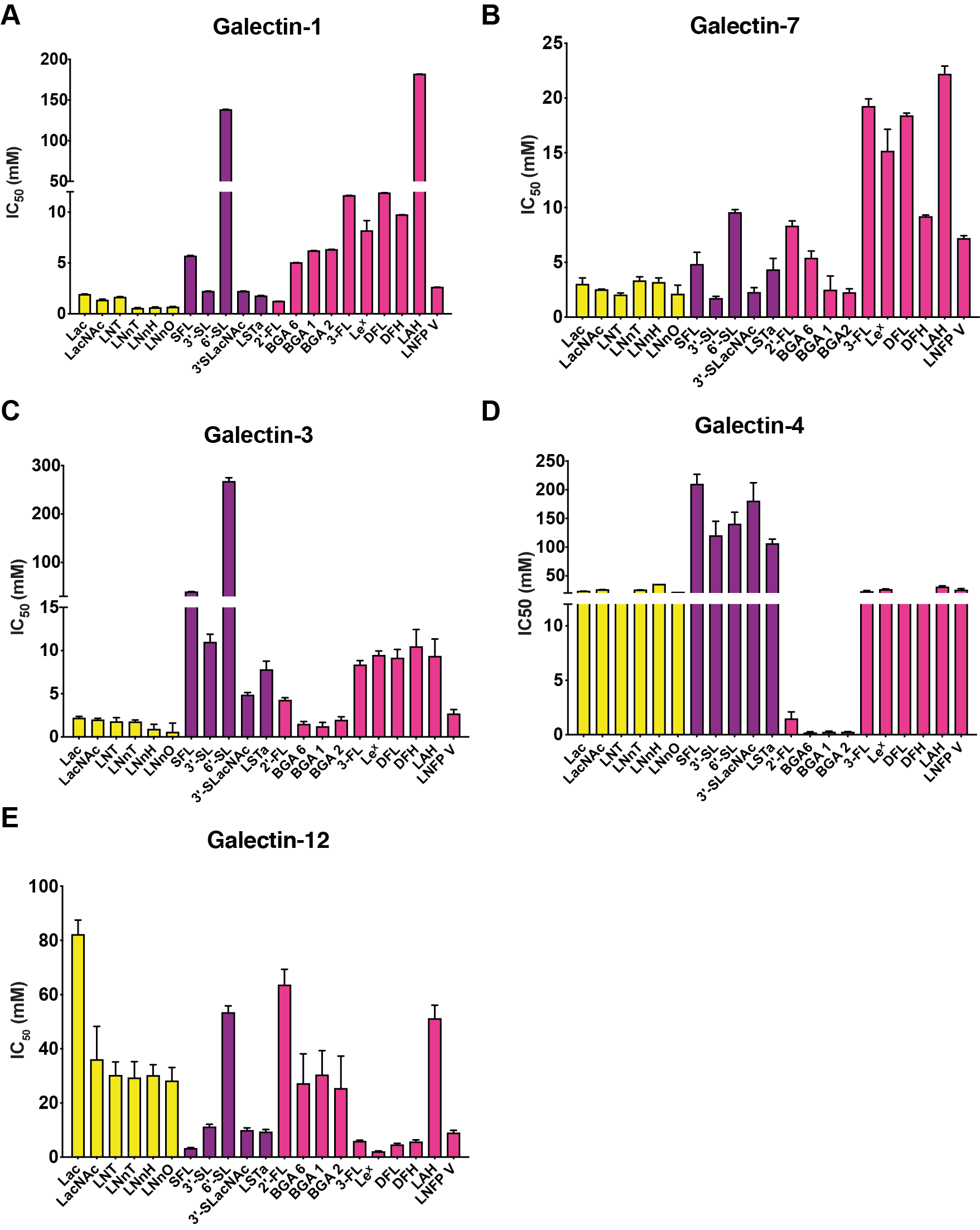
**

**Figure S1. Competitive solid phase assays for the evaluation of human galectins in interaction with HMOs and blood group glycans.** IC_50_ values for the interaction of human **(A)** Gal-1, **(B)** Gal-3, **(C)** Gal-4, **(D)** Gal-7 and **(E)** Gal-12 with a panel of 21 oligosaccharides, colored according to their structural classification: neutral non-fucosylated glycans (yellow), sialylated glycans (purple) and neutral fucosylated glycans (pink). ﻿Data presented are the mean ± S.D. from three independent experiments.

**Table S1. IC_50_ values obtained by SPAs for human Gal-1, -3, -4, -7 and -12 with tested oligosaccharides**. Data presented are the mean ± S.D. from three independent experiments.

| **Type** | **Glycan** | **IC_50_ (mM)** | | | | |
| --- | --- | --- | --- | --- | --- | --- |
|  |  | **Gal-1** | **Gal-3** | **Gal-4** | **Gal-7** | **Gal-12** |
| Neutral non-fucosylated | Lac | $1.9 \pm0.1$ | $2.2 \pm0.2$ | $23.14 \pm0.05$ | $3.0 \pm0.6$ | $82.1 \pm0.8$ |
|  | LacNAc | $1.3 \pm0.1$ | $2.0 \pm0.2$ | $25.82 \pm0.05$ | $2.49 \pm0.07$ | $36.0 \pm0.9$ |
|  | LNT | $1.6 \pm0.1$ | $1.8 \pm0.5$ | $15.1 \pm0.2$ | $2.0 \pm0.2$ | $30.1 \pm5$ |
|  | LNnT | 0$.5 \pm0.1$ | $1.8 \pm0.2$ | $25.24\pm0.05$ | $3.3 \pm0.4$ | $29.3 \pm6$ |
|  | LNnH | 0$.6 \pm0.1$ | $0.9 \pm0.6$ | $35.36 \pm0.08$ | $3.2 \pm0.4$ | $30.1 \pm4$ |
|  | LNnO | 0$.6 \pm0.1$ | $0.5 \pm0.9$ | $21.54 \pm0.03$ | $2.1 \pm0.8$ | $28.1 \pm5$ |
| Sialylated | SFL | $5.7 \pm0.1$ | $39.0 \pm0.6$ | $209.6 \pm0.8$ | $4.8 \pm0.9$ | $3.2 \pm0.4$ |
|  | 3’-SL | 2$.20 \pm0.03$ | $11.0 \pm0.9$ | $120 \pm2$ | $1.7 \pm0.2$ | $11.1 \pm1$ |
|  | 6’-SL | $138.2 \pm0.4$ | $267 \pm8$ | $140.3 \pm0.5$ | $9.5\pm0.3$ | $53.3 \pm2$ |
|  | 3’-SLacNAc | $2.20 \pm0.04$ | $4.8 \pm0.3$ | $180.3 \pm0.9$ | $2.3 \pm0.$5 | $9.8 \pm1$ |
|  | LSTa | $1.8 \pm0.1$ | $7.8 \pm0.8$ | $106 \pm8$ | $4.3 \pm0.9$ | $9.2 \pm1$ |
| Neutral fucosylated | 2’-FL | $1.23 \pm0.02$ | $4.2 \pm0.3$ | $1.5 \pm0.6$ | $8.3 \pm0.5$ | $63.5 \pm0.8$ |
|  | BGA6 | $5.02 \pm0.01$ | $1.5 \pm0.3$ | $0.2 \pm0.1$ | $5.4 \pm0.7$ | $27.1\pm1$ |
|  | BGA1 | $6.19 \pm0.01$ | $1.2 \pm0.5$ | $0.2 \pm0.1$ | $2.5 \pm0.8$ | $30.3 \pm9$ |
|  | BGA2 | $6.32 \pm0.02$ | $1.9 \pm0.4$ | $0.24 \pm0.05$ | $2.2 \pm0.4$ | $25.3 \pm12$ |
|  | 3-FL | $11.63 \pm0.02$ | $8.3 \pm0.5$ | $22.2 \pm0.9$ | $19.2 \pm0.7$ | $5.8 \pm0.5$ |
|  | Le^X^ | $8.2 \pm0.9$ | $9.5 \pm0.5$ | $26.2 \pm0.9$ | $15.1 \pm2$ | $2.0 \pm0.3$ |
|  | DFL | $11.89\pm0.02$ | $9.1 \pm0.9$ | $14.5 \pm0.7$ | $18.4 \pm0.3$ | $4.5 \pm0.6$ |
|  | DFH | $9.73 \pm0.02$ | $10.5 \pm0.7$ | $13.8 \pm3$ | $9.2 \pm0.2$ | $5.6 \pm0.8$ |
|  | LAH | $181.9 \pm0.1$ | $9.3 \pm0.6$ | $30.4 \pm2$ | $22.2 \pm0.8$ | $51.1 \pm5$ |
|  | LNFP V | $2.61 \pm0.01$ | $2.7 \pm0.5$ | $25.1 \pm0.5$ | $7.2 \pm0.3$ | $8.9 \pm1$ |

**
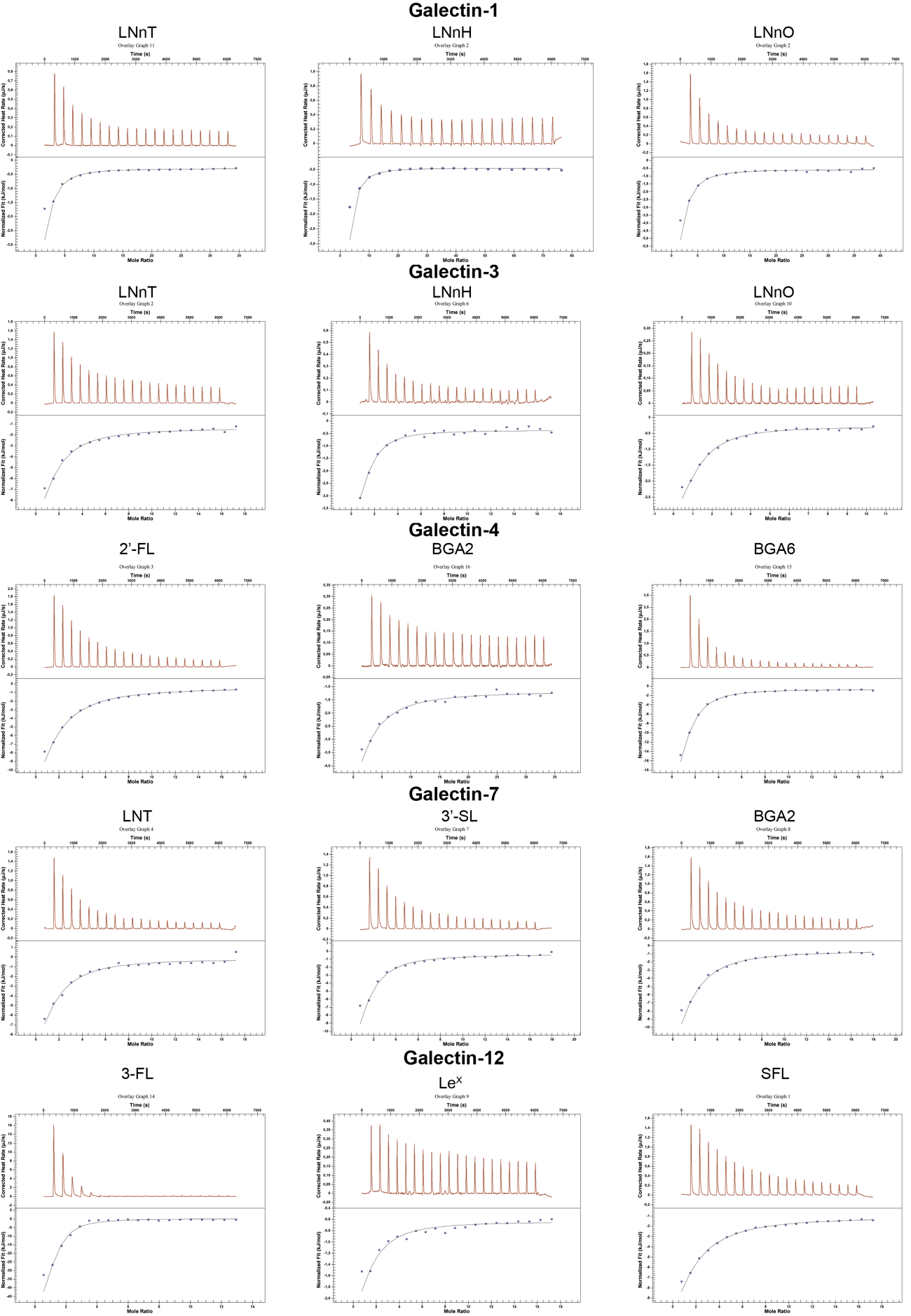
 Figure S2.** **Experimental calorimetric data by isothermal titration at 298 K**. Data obtained by ITC for human Gal-1, -3, -4, -7 and -12 with three preferentially recognized oligosaccharides.

**Table S2.** ^1^H and ^13^C chemical shifts (ppm) of 2’-fucosyllactose at 298K.

|  | Galβ | Fucα | Glcα | Glcβ |
| --- | --- | --- | --- | --- |
| H1/C1 | 4.46/100.1 | 5.24/99.3 | 5.15/91.8 | 4.57/95.9 |
| H2/C2 | 3.60/76.3 | 3.72/68.2 | 3.52/71.3 | 3.22/73.9 |
| H3/C3 | 3.80/73.6 | 3.72/69.5 | 3.73/71.22 | 3.51/74.3 |
| H4/C4 | 3.82/69.1 | 3.74/71.6 | 3.66/75.9 | 3.66/75.9 |
| H5/C5 | 3.62/75.2 | 4.17/66.9 | 3.83/70.3 | 3.41/75.3 |
| H6/C6 | 3.73,3.66/61.2 | 1.15/15.3 | 3.82,3.70/60.0 | 3.87,3.72/60.1 |

**Figure S3.** ^1^H,^13^C-HSQC spectrum of 2’-fucosyllactose recorded at 298K. Resonance assignments have been annotated.

**Table S3.** ^1^H and ^13^C chemical shifts of A type 2 antigen (BGA2) at 298K.

|  | GalNAcα | Galβ | Fucα | GlcNAcα | GlcNAcβ |
| --- | --- | --- | --- | --- | --- |
| H1/C1 | 5.11/91.3 | 4.54/100.1 | 5.28/98.6 | 5.14/90.5 | 4.66/94.9 |
| H2/C2 | 4.17/49.5 | 3.84/72.3 | 3.73/67.6 | 3.87/53.9 | 3.66/56.5 |
| H3/C3 | 3.84/67.7 | 3.92/75.7 | 3.65/69.9 | 3.78/69.3 | 3.58/72.4 |
| H4/C4 | 3.94/68.4 | 4.16/63.0 | 3.77/71.6 | 3.74/76.5 | 3.73/76.0 |
| H5/C5 | 4.14/71.0 | 3.61/75.2 | 4.25/66.8; 4.28/66.9 | 3.85/70.6 | 3.41/75.4 |
| H6/C6 | 3.71.,3.69/61.3 | 3.67,3.73/61.2 | 1.18/15.1 | 3.75,3.84/60.0 | 3.74,3.90/60.1 |
| Ac | 1.97/22.0 |  |  | 1.98/22.0 | 1.98/22.0 |

**Figure S4.** ^1^H,^13^C-HSQC spectrum of BGA2 recorded at 298K. Resonance assignments have been annotated.

**Table S4.** ^1^H and ^13^C chemical shifts of A type 6 antigen (BGA6) at 298K.

|  | GalNAcα | Galβ | Fucα | Glcα | Glcβ |
| --- | --- | --- | --- | --- | --- |
| H1/C1 | 5.05/91.3 | 4.46/100.0 | 5.22/98.7 | 5.11/91.9 | 4.51/95.9 |
| H2/C2 | 4.11/49.5 | 3.78/72.4 | 3.66/67.8 | 3.47/71.3 | 3.17/73.9 |
| H3/C3 | 3.78/67.8 | 3.86/75.7 | 3.59/69.9 | 3.66/71.3 | 3.44/74.3 |
| H4/C4 | 3.87/68.5 | 4.10/63.1 | 3.71/71.6 | 3.60/71.3 | 3.61/75.9 |
| H5/C5 | 4.09/71.1 | 3.53/75.1 | 4.24/67.0;4.20/66.9 | 3.77/70.4 | 3.34/75.4 |
| H6/C6 | 3.63,3.64/61.2 | 3.60,3.68/61.2 | 1.17/15.1 | 3.67,3.77/60.0 | 3.83,3.64/60.1 |
| NAc | 1.96/21.9 |  |  |  |  |

**Figure S5.** ^1^H,^13^C-HSQC spectrum of BGA6 recorded at 298K. Resonance assignments have been annotated.

**Abbreviations:**

Lac, lactose; LacNAc, *N*-acetyllactosamine; LNnT, lacto-*N*-neotetraose; LNT, lacto-*N*-tetraose; LNnH, lacto-*N*-neohexaose; LNnO, lacto-*N*-neooctaose; SFL, 3’-sialyl-3-fucosyllactose; 3′-SL, 3′-sialyllactose; 6′-SL, 6′-sialyllactose; 3’-SLacNAc, 3’-sialyl-*N*-acetyllactosamine; LSTa, sialyl-lacto-*N*-tetraose a; 2′-FL, 2′-fucosyllactose; BGA6, blood group A antigen 6; BGA1, blood group A antigen 1; BGA2, blood group A antigen 2; 3-FL, 3-fucosyllactose; Le^X^, Lewis X trisaccharide; DFL, 2′,3-difucosyllactose; DFH, 3,3-difucosylhexaose (Lewis X hexaose); LAH, Lewis A antigen hexaose; LNFPV, lacto-*N*-fucopentaose V.
